# Dual tumor microenvironment-responsive albumin nanoplatform integrates conditional PROTAC activation with starvation and ferroptosis for synergistic cancer therapy

**DOI:** 10.1186/s12951-026-04266-9

**Published:** 2026-03-09

**Authors:** Lingting Lin, Binyu Chen, Yourui Yang, Ruocheng Deng, Wenfei Niu, Jian Liu, Wei Xu, Hua Li

**Affiliations:** 1 Institute of Structural Pharmacology & TCM Chemical Biology, Fujian Key Laboratory of Chinese Materia Medica, College of Pharmacy, Fujian University of Traditional Chinese Medicine, No. 1, Qiuyang Road, Fuzhou, Fujian 350122 China; 2 Fujian Health College, Fuzhou, 350101 China

**Keywords:** Ferroptosis, PROTAC, Hypoxia, Albumin nanoparticle, Glucose oxidase, Starvation therapy

## Abstract

**Background:**

Proteolysis-targeting chimeras (PROTACs) have emerged as a promising cancer therapeutic approach by targeting protein degradation to address undruggable targets and drug resistance associated with conventional therapies, yet their clinical translation is hindered by poor solubility and non-specific toxicity. Additionally, tumor heterogeneity and biological complexity frequently limit the efficacy of monotherapies, necessitating the development of multifunctional delivery systems.

**Results:**

We engineered a dual microenvironment-responsive albumin to integrate conditional PROTAC activation with metabolic starvation and ferroptosis for synergistic antitumor efficacy within a unified therapeutic cascade. Ferrocene (Fc)-modified human serum albumin (HSA) via coupling chemistry was electrostatically complexed with glucose oxidase (GOD) and co-assembled with an azobenzene (AZO)-caged ARV-771 prodrug to yield HSA-Fc-GOD@ARV-771(AZO) nanoparticles that exhibited pH-triggered disassembly in acidic tumor environments. the released GOD consumes glucose and oxygen to generate hydrogen peroxide while inducing metabolic starvation and exacerbating hypoxia. The intensified hypoxia triggers nitroreductase to cleave the prodrug linker and release the active ARV-771, which subsequently degrades bromodomain containing protein 4. This degradation directly suppresses tumor cell proliferation and downregulates glutathione peroxidase 4 expression to sensitize cancer cells to oxidative damage. Concurrently, the ferrocene component converts the generated hydrogen peroxide into hydroxyl radicals via the Fenton reaction to amplify lipid peroxidation and ferroptotic cell death. The nanoplatform suppressed lung cancer cell viability to 10.8% in vitro and achieved 94.3% tumor growth inhibition in xenograft models without observable systemic toxicity.

**Conclusions:**

This study demonstrates a precision therapeutic paradigm. It exploits tumor microenvironment characteristics to couple conditional drug activation with starvation and ferroptosis. This strategy offers a translatable framework for next generation’s cancer treatment.

**Graphical Abstract:**

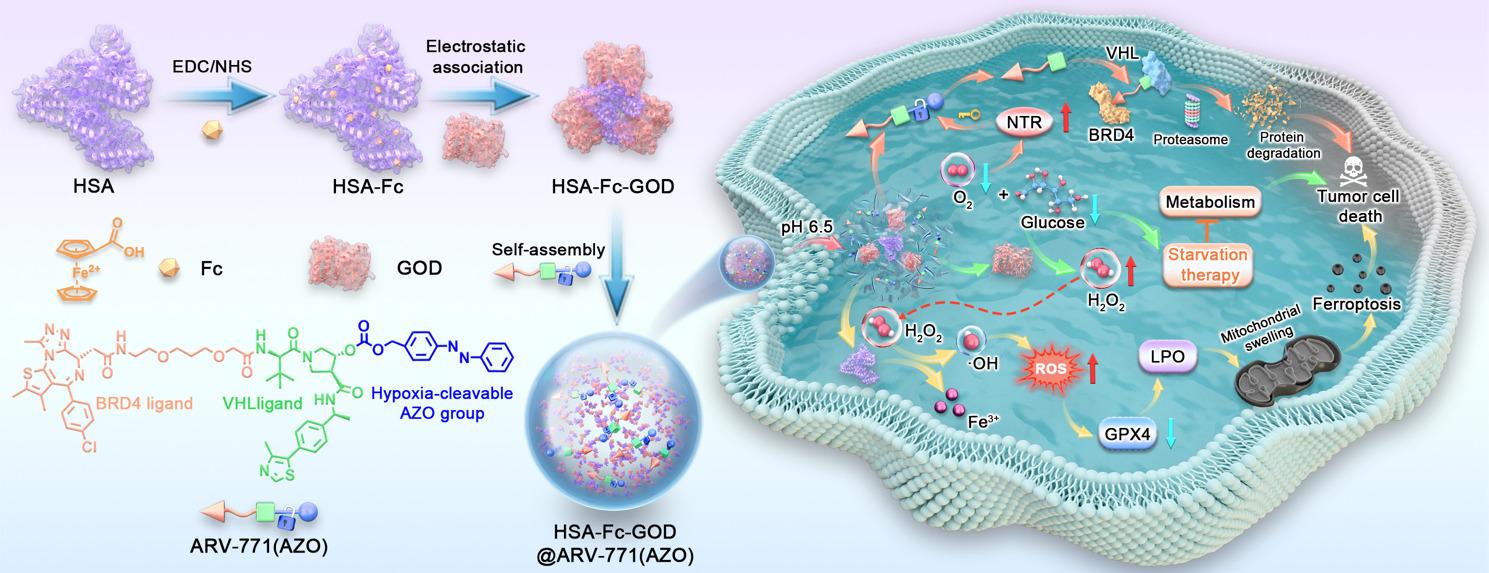

**Supplementary Information:**

The online version contains supplementary material available at 10.1186/s12951-026-04266-9.

## Introduction

Lung cancer constitutes the primary cause of cancer-related mortality worldwide [1–3]. While standard chemotherapies based on platinum agents and taxanes provide clinical benefit, their durability is frequently undermined by systemic toxicity and acquired resistance. Tumor cells often evade treatment through efflux pumps that actively expel therapeutic agents or by activating compensatory signaling pathways that bypass the original drug targets [4]. These adaptations underscore the critical need for strategies that eliminate cancer cells through mechanisms distinct from apoptosis [5, 6]. Furthermore, tumor heterogeneity and metabolic adaptation limit the efficacy of single-modality treatments and necessitate delivery systems capable of coordinating multiple distinct vulnerabilities within the same lesion [7, 8].

Targeted protein degradation (TPD) has emerged as a transformative therapeutic paradigm, with proteolysis-targeting chimeras (PROTACs) representing the most advanced class [9, 10]. Unlike traditional inhibitors that merely block protein function, PROTACs such as ARV-771 induce the ubiquitination and subsequent proteasomal destruction of their targets [11]. ARV-771 specifically eliminates bromodomain-containing protein 4 (BRD4), a key oncogenic transcription regulator [12, 13]. This catalytic turnover allows a single PROTAC molecule to destroy multiple target proteins sequentially. However, poor water solubility and limited membrane permeability act as significant barriers to the clinical translation of many PROTAC candidates [14–16].

Recent advances utilizing lipid nanoparticles and polymeric micelles have improved the systemic delivery of PROTACs [17, 18]. Yet many current carriers serve primarily as inert transport vehicles that solve formulation issues without contributing to the therapeutic mechanism. An alternative strategy involves coupling delivery with microenvironment reprogramming where the carrier itself actively amplifies the payload activity. Albumin serves as an ideal scaffold for this purpose given its biocompatibility and established clinical use [19]. Albumin-based nanocarriers can achieve favorable tumor accumulation through the enhanced permeability and retention effect as well as receptor-mediated transport pathways involving gp60 and SPARC interactions [20].

Ferroptosis offers a regulated cell death pathway distinct from apoptosis that remains effective even in cells utilizing classical anti-apoptotic mutations [21]. This process is driven by iron-dependent lipid peroxidation (LPO) and becomes lethal when lipid hydroperoxides accumulate beyond the cellular detoxification capacity [22, 23]. Such a mechanism aligns well with catalytic cascade designs because LPO can be amplified by sustaining local oxidant supplies [24]. A major limitation is that tumors often lack sufficient endogenous hydrogen peroxide to fuel iron-mediated oxidation [25]. To address this limitation, glucose oxidase (GOD) has been incorporated into nanoplatforms where it consumes tumor glucose and generates H_2_O_2_ in situ, thereby amplifying Fenton reactions while simultaneously inducing metabolic starvation [26–29].

Cancer cells defend against ferroptosis through antioxidant systems relying on glutathione peroxidase 4 (GPX4) to neutralize lipid hydroperoxides [30]. Since BRD4 regulates the transcription of antioxidant defense genes, its suppression reduces GPX4 expression and lowers the threshold for ferroptotic death [31, 32]. Our design leverages this specific dependency. Ferroptosis is not treated here as an incidental additive effect but as the execution step that converts enzyme-generated hydrogen peroxide into lethal membrane damage. Simultaneous BRD4 degradation compromises the GPX4 barrier and renders the cell unable to buffer this oxidative stress. This creates a synthetic lethal interaction that mechanistically distinct inputs like chemotherapy or radiotherapy would not achieve.

We acknowledge that ferroptosis-inducing agents are largely preclinical and carry potential risks of systemic oxidative injury. Therefore, we position this work as a proof-of-concept for microenvironment-controlled cascade therapy. The clinical success of albumin-bound paclitaxel supports the translational potential of albumin as a carrier platform [33]. Furthermore, relying on dual triggers prevents off-target activation since hypoxia can occur in non-malignant ischemic tissues. Our strategy requires the convergence of specific metabolic conditions to maximize safety.

We engineered a multifunctional nanoplatform comprising ferrocene-modified human serum albumin (HSA-Fc), GOD, and an azobenzene (AZO)-caged ARV-771 prodrug. The resulting construct, designated HSA-Fc-GOD@ARV-771(AZO), operates as a connected therapeutic sequence rather than a combination of independent agents. Integrated GOD consumes intratumoral glucose to initiate starvation while simultaneously generating H₂O₂ and depleting local oxygen. The resulting hypoxia triggers reduction of the AZO linker to release active ARV-771. The released PROTAC degrades BRD4, which both inhibits tumor proliferation directly and downregulates antioxidant defenses to sensitize cells to oxidative stress. The generated H₂O₂ is converted by ferrocene into hydroxyl radicals (•OHs) that drive ferroptosis. This design supports a strategy where starvation therapy, PROTAC technology, and ferroptosis converge to produce a collaborative antitumor effect that simple additive combinations may not achieve.

## Materials and methods

### Materials

HSA and GOD were purchased from Aladdin Biochemical Technology Co., Ltd. (Shanghai, China). Ferrocenecarboxylic acid and ARV-771 were obtained from BiDe Pharmaceutical Technology Co., Ltd. (Shanghai, China). [Ru(dpp)₃]Cl₂, 3-MeOARH-NTR, DCFH-DHA, Ferro Orange, C11 BODIPY 581/591, and Mito-Tracker were acquired from LABLEAD Co., Ltd. (Beijing, China). Antibodies against BRD4 and GPX4 were sourced from Abclonal Biotechnology Co., Ltd. (Wuhan, China). Hoechst 33,258, Ham’s F12K medium, trypsin-ethylenediaminetetraacetic acid, and fetal bovine serum were purchased from Gibco-BRL (Burlington, Canada). All other reagents were obtained from J&K Scientific, Ltd. and used as received.

### Synthesis of HSA-Fc conjugate

Ferrocenecarboxylic acid (25.3 mg, 0.11 mmol), 1-ethyl-3-(3-dimethylaminopropyl)carbodiimide hydrochloride (EDC, 19.2 mg, 0.10 mmol), and N-hydroxysuccinimide (NHS, 12.7 mg, 0.11 mmol) were dissolved in anhydrous dimethyl sulfoxide (DMSO, 5 mL) and reacted at room temperature for 1 h to activate the carboxyl group. The resulting NHS ester was added to a solution of HSA (72 mg in 10 mL PBS, pH 7.4) and stirred overnight at room temperature. The mixture was centrifuged (14,000 rpm, 10 min) to remove aggregates, and unreacted ferrocenecarboxylic acid was eliminated by three cycles of ultrafiltration (MWCO = 10 kDa). The purified HSA-Fc conjugate was stored at 4 °C.

### Synthesis of ARV-771(AZO)

Compound 1: Nitrosobenzene (2.39 g, 22.3 mmol) was added to a solution of 4-aminobenzyl alcohol (2.50 g, 20.3 mmol) in glacial acetic acid (100 mL) and stirred overnight at room temperature. After solvent removal under reduced pressure, the residue was extracted with ethyl acetate, washed sequentially with saturated NaHCO₃ solution and brine, and dried over anhydrous MgSO₄. Purification by silica gel chromatography (petroleum ether/ethyl acetate, 4:1 → 2:1, v/v) afforded Compound 1 as a white solid (3.03 g, 85.3%). ^1^H NMR (400 MHz, DMSO-*d6*) δ 7.96–7.82 (m, 5 H), 7.67–7.50 (m, 4 H), 5.40 (t, J = 5.6 Hz, 2 H), 4.61 (d, J = 5.5 Hz, 1H).

Compound 2: Compound 1 (80 mg, 0.40 mmol) was dissolved in anhydrous dichloromethane (50 mL) with anhydrous pyridine (0.1 mL, 1.2 mmol). 4-Nitrophenyl chloroformate (160 mg, 0.8 mmol) was added, and the mixture was stirred for 2 h under nitrogen atmosphere. The reaction mixture was diluted with dichloromethane, washed with 1 M aqueous citric acid, dried over anhydrous MgSO₄, and concentrated under reduced pressure. Purification by silica gel chromatography (dichloromethane/hexane, 4:1 → 2:1, v/v) afforded Compound 2 as a pale yellow solid (56.2 mg, 38.3%). ^1^H NMR (400 MHz, DMSO-*d6*) δ 8.42–8.28 (m, 2 H), 8.03–7.84 (m, 4 H), 7.77–7.69 (m, 2 H), 7.67–7.53 (m, 5 H), 5.43 (s, 2 H).

ARV-771(AZO): Compound 2 (150 mg, 0.40 mmol), ARV-771 (100 mg, 0.10 mmol), 4-dimethylaminopyridine (DMAP, 12.2 mg, 0.10 mmol), and triethylamine (40 µL) were dissolved in anhydrous dichloromethane (30 mL) and stirred at room temperature for 24 h under nitrogen atmosphere. After solvent removal under reduced pressure, the crude product was purified by silica gel chromatography (dichloromethane/methanol, 100:1 → 30:1, v/v) to afford ARV-771(AZO) as a pale yellow solid (176.2 mg, 32.4%). ^1^H NMR (400 MHz, DMSO-*d6*) δ 9.05 (s, 1H), 8.99 (s, 3 H), 8.79 (s, 1H), 8.50 (d, J = 7.6 Hz, 15 H), 8.28 (t, J = 5.6 Hz, 7 H), 7.95–7.87 (m, 6 H), 7.60 (ddd, J = 11.5, 7.2, 4.4 Hz, 8 H), 7.50–7.34 (m, 7 H), 6.90–6.62 (m, 2 H), 5.76 (s, 3 H), 5.24 (d, J = 20.5 Hz, 5 H), 4.91 (t, J = 7.2 Hz, 15 H), 4.54–4.42 (m, 6 H), 4.37 (s, 1H), 4.03 (d, J = 12.1 Hz, 5 H), 3.90 (s, 2 H), 3.85–3.70 (m, 15 H), 3.61–3.38 (m, 8 H), 3.32–3.07 (m, 2 H), 2.67 (s, 4 H), 2.58 (s, 5 H), 2.45 (s, 6 H), 2.39 (s, 6 H), 2.07–1.96 (m, 13 H), 1.91 (s, 4 H), 1.77 (p, J = 6.2 Hz, 3 H), 1.60 (d, J = 3.5 Hz, 4 H), 1.47 (d, J = 6.9 Hz, 5 H), 1.40–1.20 (m, 2 H), 1.13–0.97 (m, 2 H), 0.94 (d, J = 13.4 Hz, 3 H).

Compound 3: 4-(2-Phenylethyl)benzenemethanol (BZM, 80 mg, 0.40 mmol) was reacted with 4-nitrophenyl chloroformate (160 mg, 0.8 mmol) in anhydrous dichloromethane (50 mL) with pyridine (0.1 mL, 1.2 mmol) for 2 h. The mixture was worked up and purified by silica gel chromatography (dichloromethane: hexane, 4:1 → 2:1, v/v) to obtain Compound 3 as a white solid (76.2 mg, 87.5%). ^1^H NMR (400 MHz, DMSO-*d*6) δ 8.32 (d, J = 9.0 Hz, 2 H), 7.58 (d, J = 9.0 Hz, 2 H), 7.37 (d, J = 7.8 Hz, 2 H), 7.31–7.16 (m, 7 H), 5.27 (s, 2 H), 2.90 (s, 4 H).

ARV-771(BZM): Compound 3 (150 mg, 0.40 mmol) was reacted with ARV-771 (100 mg, 0.10 mmol), DMAP (12.2 mg, 0.10 mmol), and triethylamine (40 µL) in anhydrous dichloromethane (30 mL) for 24 h. Purification by silica gel chromatography (dichloromethane: methanol, 100:1 → 30:1, v/v) yielded ARV-771(BZM) (96.2 mg, 43.7%). ^1^H NMR (400 MHz, DMSO-*d6*) δ 8.99 (s, 1H), 8.48 (d, J = 7.7 Hz, 1H), 8.28 (s, 1H), 7.76–7.64 (m, 2 H), 7.51–7.33 (m, 12 H), 7.27 (dd, J = 14.1, 7.1 Hz, 7 H), 7.27–7.12 (m, 5 H), 5.10 (s, 2 H), 4.55–4.40 (m, 4 H), 4.22 (t, J = 6.5 Hz, 3 H), 3.92 (d, J = 2.9 Hz, 2 H), 3.58 (s, 1H), 3.51 (dt, J = 18.0, 6.5 Hz, 5 H), 3.42 (t, J = 5.6 Hz, 2 H), 3.31–3.18 (m, 3 H), 2.87 (s, 4 H), 2.59 (s, 3 H), 2.45 (s, 4 H), 2.40 (s, 3 H), 1.82–1.75 (m, 2 H), 1.63 (d, J = 13.1 Hz, 7 H), 1.43–1.32 (m, 9 H), 1.32–1.23 (m, 13 H), 0.97 − 0.89 (m, 9 H).

### Preparation of HSA-Fc-GOD@ARV-771(AZO) nanoparticles

HSA-Fc (10 mg) and GOD (0.1 mL of a 10 mg/mL solution in PBS) were mixed in PBS (1 mL, pH 7.4) for 30 min at room temperature, followed by ultrafiltration (10,000 MWCO, 4000 rpm, 10 min) to form the HSA-Fc-GOD complex. A solution of ARV-771(AZO) in methanol (20 µL of 50 mg/mL) was added dropwise, and the mixture was stirred overnight at room temperature to induce self-assembly. The resulting nanoparticles were purified by centrifugation (5000 rpm, 5 min at 4 °C) and ultrafiltration (MWCO = 100 kDa) to remove unencapsulated drug and methanol. HSA-Fc-GOD@ARV-771(BZM) nanoparticles were prepared using an identical procedure.

### Characterization

X-ray photoelectron spectroscopy (XPS) was performed on a Thermo Scientific K-Alpha system. Nanoparticle morphology was observed using scanning electron microscopy (SEM; Hitachi Regulus8100) and transmission electron microscopy (TEM; FEI TECNAI G2F20). ¹H NMR spectra were recorded on a Bruker 400 MHz spectrometer. UV-vis-NIR absorption spectra were obtained with a Shimadzu UV-2700 spectrophotometer. Fe²⁺ concentrations were quantified by inductively coupled plasma-mass spectrometry (ICP-MS; Thermo Fisher Scientific iCAP7400). Dynamic light scattering (DLS) measurements of hydrodynamic diameter and zeta potential were conducted using a Malvern Zetasizer Nano ZS instrument at 25 °C.

### Hemolysis assay

A diluted suspension of murine red blood cells (0.2 mL) was mixed with 0.8 mL of PBS (negative control), distilled water (positive control), or HSA-Fc-GOD@ARV-771(AZO) solutions at various concentrations. After 2 h of incubation at 37 °C, the samples were centrifuged, and the absorbance of the supernatant was measured at 541 nm. The hemolysis percentage was calculated using the following equation: [(A_sample_ - A_negative_) / (A_positive_ - A_negative_)] × 100%.

### Drug release studies

HSA-Fc-GOD@ARV-771(AZO) solutions (1 mg/mL) were placed in dialysis bags (MWCO = 3.5 kDa) and dialyzed against PBS (pH 6.5 or 7.4) containing Na₂S₂O₄ (10 mM) to simulate NTR activity at 37 °C with gentle shaking (100 rpm). At designated time points, aliquots of the external medium were withdrawn for analysis by UV-vis absorption spectroscopy at 254 nm and replaced with an equal volume of fresh buffer.

### Cellular assays

A549 cells were seeded in confocal dishes (1.5 × 10⁵ cells/dish) and incubated for 24 h to allow attachment. Cells were then treated under hypoxia (1% O₂) with medium (control), ARV-771(BZM), ARV-771(AZO), HSA-Fc-GOD, HSA-Fc-GOD@ARV-771(BZM), or HSA-Fc-GOD@ARV-771(AZO) ([HSA-Fc-GOD] = 5.28 µg/mL, [ARV-771(AZO)] = 2.45 µg/mL) for 12 h.

Oxygen, NTR and H_2_O_2_ detection: Following treatment, cells were incubated with [Ru(dpp)₃]Cl₂ (4.0 µg/mL), 3-MeOARH-NTR (6.0 µg/mL), or H_2_O_2_ probe (1.0 µg/mL) for 20 min at 37 °C in the dark.

Fe(II), ROS, and lipid peroxide detection: Cells were incubated with Ferro Orange (0.68 µg/mL), DCFH-DHA (5.0 µg/mL), or C11 BODIPY 581/591 (2.3 µg/mL) for 30 min.

Mitochondrial Membrane Potential: Cells were stained with Mito-Tracker (0.1 µg/mL) for 30 min.

For all imaging, cells were counterstained with Hoechst 33,258 and observed by confocal laser scanning microscopy (CLSM).

### TEM of mitochondria

A549 cells (1 × 10⁷ cells/dish) were treated with HSA-Fc-GOD@ARV-771(AZO) ([HSA-Fc-GOD] = 5.28 µg/mL) or culture medium alone for 12 h under hypoxic conditions (1% O₂). Cells were harvested, washed with PBS, and fixed with 2.5% glutaraldehyde in 0.1 M phosphate buffer (pH 7.4) for 2 h at 4 °C, post-fixed with 1% osmium tetroxide for 1 h, dehydrated through a graded ethanol series (30%, 50%, 70%, 90%, 95%, and 100%), and embedded in epoxy resin. Ultrathin Sect.  (70 nm) were cut using an ultramicrotome, stained with 2% uranyl acetate for 15 min and Reynolds’ lead citrate for 10 min before TEM imaging on a FEI TECNAI G2F20 microscope operated at 200 kV.

### Western blot analysis

A549 cells (5.4 × 10⁶ cells/dish) were treated with culture medium alone, ARV-771(BZM), ARV-771(AZO), HSA-Fc-GOD, HSA-Fc-GOD@ARV-771(BZM), or HSA-Fc-GOD@ARV-771(AZO) ([HSA-Fc-GOD] = 5.28 µg/mL, [ARV-771(AZO)] = 2.45 µg/mL) for 24 h under hypoxic conditions (1% O₂), and total cellular protein was extracted using RIPA lysis buffer supplemented with protease inhibitors. Equal amounts of protein (30 µg per lane) were resolved by SDS-PAGE (10% gel), transferred to PVDF membranes, and blocked with 5% non-fat milk in TBST for 1 h at room temperature. Membranes were incubated with primary antibodies against GPX4 (1:1000 dilution) or BRD4 (1:1000 dilution) overnight at 4 °C, followed by HRP-conjugated secondary antibodies (1:5000 dilution, 1 h at room temperature). Protein bands were visualized using a ChemiDoc XRS+ system with enhanced chemiluminescence substrate.

### In vitro cytotoxicity assays

A549 cells (5 × 10³ cells/well in 96-well plates) were treated with various concentrations of ARV-771(AZO) or ARV-771(BZM) (0.5–16 µM) for 48 h under normoxic (21% O₂) or hypoxic (1% O₂) conditions. Cell viability was assessed using the MTT assay according to the manufacturer’s protocol.

For HSA-Fc-GOD@ARV-771(AZO) cytotoxicity evaluation, A549 cells (5 × 10³ cells/well) were treated with ARV-771(BZM), ARV-771(AZO), HSA-Fc-GOD, HSA-Fc-GOD@ARV-771(BZM), or HSA-Fc-GOD@ARV-771(AZO) at various concentrations (equivalent to 0.5–16 µM ARV-771) for 48 h under hypoxic conditions (1% O₂), followed by MTT assay. Absorbance was measured at 570 nm using a microplate reader, and cell viability was calculated as a percentage relative to untreated control cells.

### In vivo antitumor studies

Female BALB/c nude mice (6–8 weeks old, 18–22 g) were subcutaneously injected in the right flank with A549 cells (5 × 10^6^ cells in 100 µL PBS). When tumors reached approximately 60 mm³, mice (*n* = 5/group) were randomly assigned to six groups using a random number generator: (ⅰ) control (PBS), (ⅱ) ARV-771(BZM), (ⅲ) ARV-771(AZO), (ⅳ) HSA-Fc-GOD, (ⅴ) HSA-Fc-GOD@ARV-771(BZM), and (ⅵ) HSA-Fc-GOD@ARV-771(AZO). Treatments ([HSA-Fc-GOD] = 10 mg/kg, [ARV-771(AZO)] = 4.65 mg/kg in 100 µL PBS) were administered intravenously via the tail vein every three days for a total of 7 doses. Tumor volume was calculated using the formula V = (length × width²)/2, and body weight was monitored every three days. After 21 days, mice were euthanized, and tumors and major organs (heart, liver, spleen, lungs, and kidneys) were harvested for histological analysis. Tumor tissues were fixed in 10% neutral buffered formalin, embedded in paraffin, sectioned at 5 μm thickness, and stained with hematoxylin and eosin (H&E) or processed for immunohistochemistry using antibodies against BRD4 and GPX4.

### Statistical analysis

Quantitative data are presented as the mean ± standard deviation (SD) of at least three independent experiments. Statistical significance was assessed using Student’s t-test for two-group comparisons or one-way analysis of variance (ANOVA) with Tukey’s post hoc test for multiple group comparisons using GraphPad Prism 9.5 software. A p-value less than 0.05 was considered statistically significant. Significance levels are indicated as *p < 0.05, **p < 0.01, and ***p < 0.001.

## Results and discussion

### Construction and physicochemical characterization of the nanoplatform

**Fig. 1:**
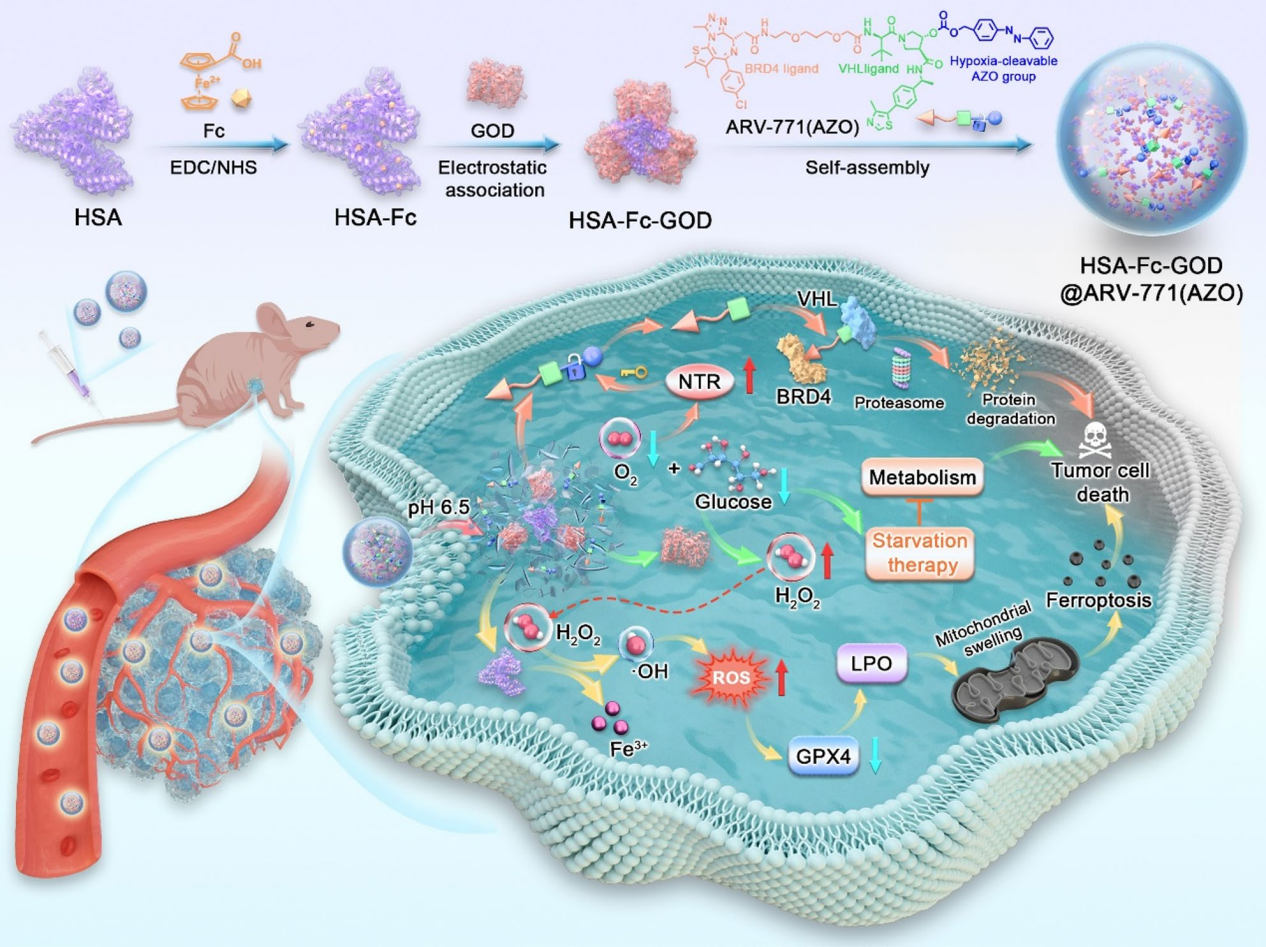
Schematic illustration of the pH- and hypoxia-responsive HSA-Fc-GOD@ARV-771(AZO) nanoplatform engineered for synergistic cancer therapy through integrated starvation, ferroptosis induction, and PROTAC-mediated protein degradation. Acidic tumor pH triggers nanoparticle disassembly and GOD release, which depletes glucose and oxygen while generating H_2_O_2_. Exacerbated hypoxia upregulates NTR expression, catalyzing AZO cleavage to liberate ARV-771 for BRD4 degradation. Concurrently, ferrocene-mediated Fenton reactions convert H_2_O_2_ into •OHs, triggering LPO and ferroptotic cell death sensitized by GPX4 downregulation

**Fig. 2:**
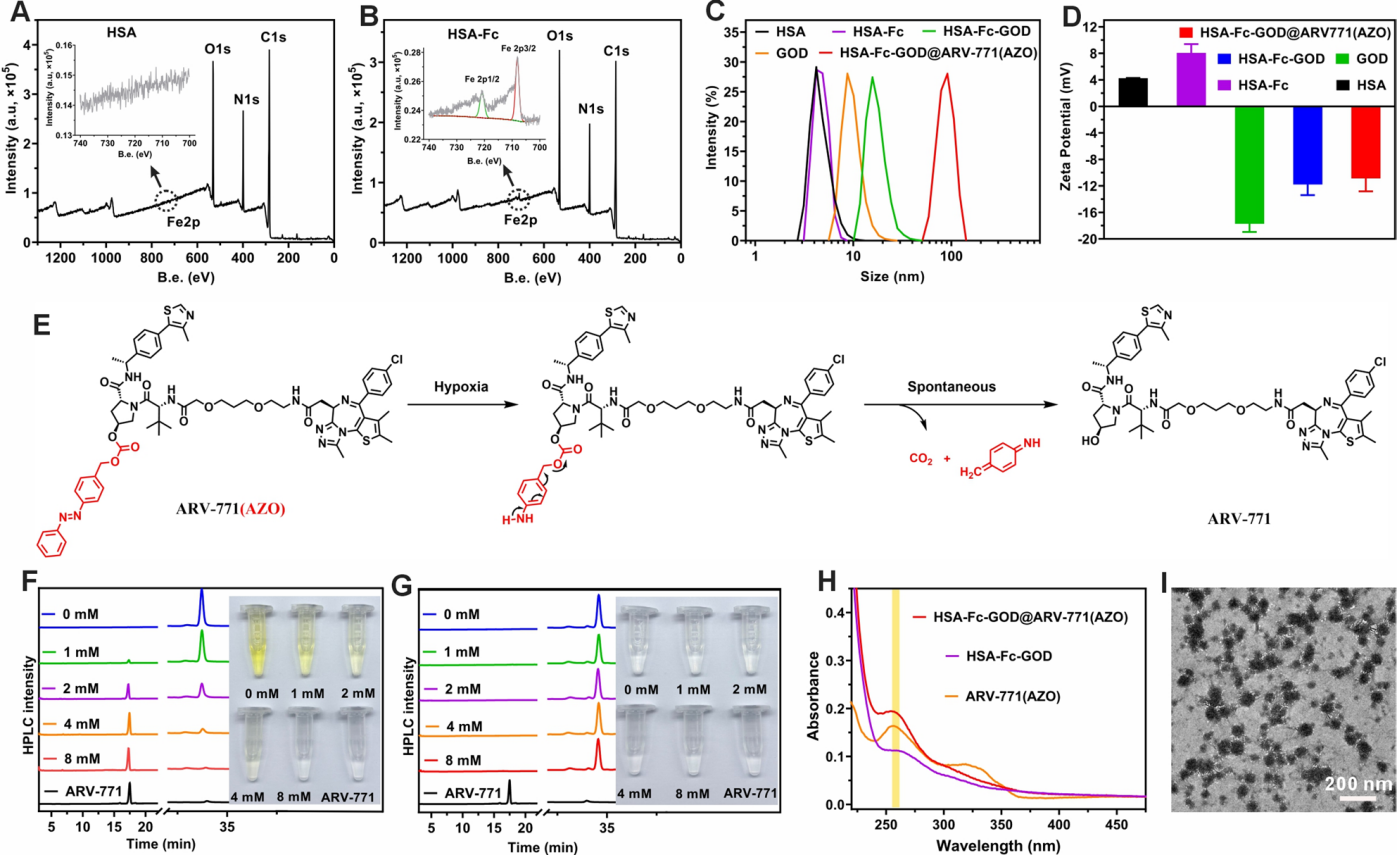
Construction and physicochemical characterization of pH- and hypoxia-activatable HSA-Fc-GOD@ARV-771(AZO) nanoparticles. (**A, B**) XPS survey spectra of native HSA (A) and Fc-modified HSA-Fc (**B**), with insets displaying high-resolution Fe 2p spectra confirming successful conjugation. (**C, D**) Dynamic light scattering measurements of hydrodynamic diameter (**C**) and zeta potential (**D**) during stepwise nanoplatform assembly. (**E**) Proposed mechanism for hypoxia-triggered ARV-771 release via NTR-mediated AZO cleavage. (**F, G**) HPLC chromatograms demonstrating concentration-dependent ARV-771 release from ARV-771(AZO) (**F**) compared with the non-cleavable control ARV-771(BZM) (**G**) following incubation with varying Na₂S₂O₄ concentrations as NTR surrogate. (**H**) UV-vis-NIR absorption spectra of HSA-Fc-GOD before and after ARV-771(AZO) encapsulation. (**I**) Representative TEM image of HSA-Fc-GOD@ARV-771(AZO) nanoparticles. Scale bar, 100 nm

We constructed a multifunctional nanoplatform, HSA-Fc-GOD@ARV-771(AZO), designed to integrate ferroptosis induction, metabolic disruption, and PROTAC within a single hypoxia-responsive system (Fig. 1). The synthesis began with chemical conjugation of ferrocene to human serum albumin using EDC/NHS coupling chemistry. X-ray photoelectron spectroscopy identified distinct Fe 2p3/2 and Fe 2p1/2 signals at 708.5 eV and 722.4 eV which were absent in the native protein. The peak positions correspond to ferrocene iron in the Fe(II) state while the integrated area ratio aligns with the theoretical 2 to 1 spin-orbit splitting value [34]. The modification of Fc did not alter the hydrodynamic size of HSA but increased the zeta potential from + 4 mV to + 10 mV (Fig. 2C, D), thereby facilitating subsequent electrostatic association with the negatively charged enzyme GOD. The surface potential reversed to − 11.8 mV following enzyme binding. This negative shift indicates that the enzyme is not fully encapsulated within a core but rather forms a composite surface layer with exposed domains.

To endow the PROTAC with tumor-selective activation, an AZO linker was introduced at the hydroxyl position of ARV-771’s VHL-binding moiety to yield a hypoxia-cleavable prodrug, ARV-771(AZO). A non-cleavable benzylmethyl analogue designated (ARV-771(BZM)) was prepared as a negative control. All intermediates and final products were structurally verified by ¹H NMR spectroscopy and mass spectrometry (Fig. S1-7). Hypoxia-triggered cleavage was evaluated using Na₂S₂O₄ as an NTR mimetic (Fig. 2E) [35]. HPLC analysis revealed that the peak corresponding to ARV-771(AZO) decreased progressively with increasing Na₂S₂O₄ concentration, accompanied by the appearance of a new peak consistent with free ARV-771 (Fig. 2F). This transformation was accompanied by a visible color change of the solution from yellow to colorless. In contrast, the control compound ARV-771 (BZM) exhibited no detectable change (Fig. 2G), thereby confirming that the AZO linker underwent selective reductive cleavage under hypoxia-mimicking conditions.

**Fig. 3:**
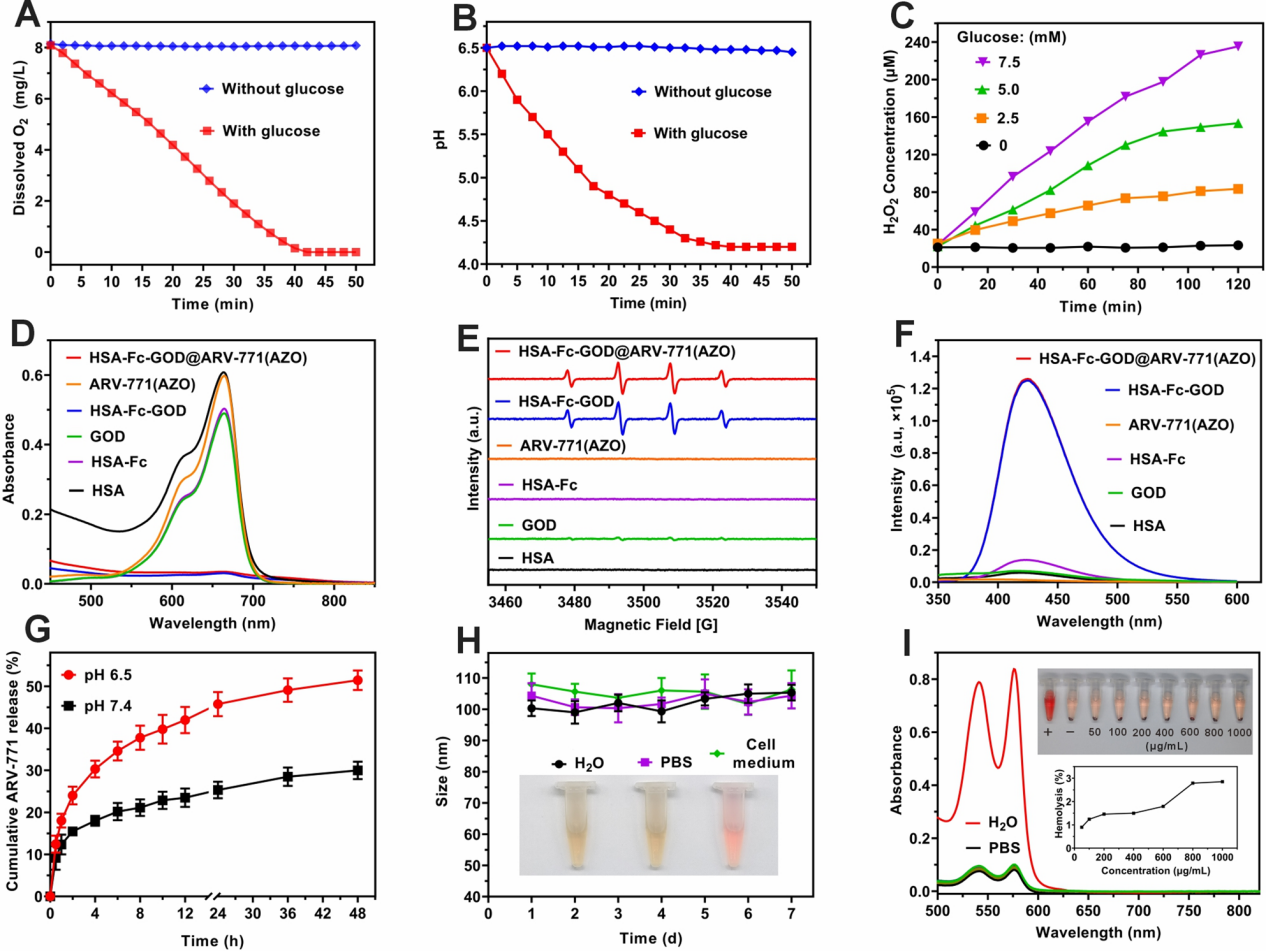
Functional validation of coupled enzymatic cascade, dual-responsive drug release, and biocompatibility assessment. (**A, B**) Time-dependent changes in dissolved oxygen concentration (**A**) and pH (**B**) of HSA-Fc-GOD@ARV-771(AZO) solution in the presence or absence of glucose (2 mg/mL). (C) Glucose concentration-dependent H_2_O_2_ generation by HSA-Fc-GOD@ARV-771(AZO) nanoparticles. (**D**) UV-vis spectral analysis of MB degradation following incubation with HSA-Fc-GOD@ARV-771(AZO) and individual components. (**E**, **F**) ESR spectra of DMPO/•OH adducts (**E**) and fluorescence spectra of terephthalic acid/•OH complexes (**F**). (**G**) pH-dependent and hypoxia-responsive ARV-771 release profiles from HSA-Fc-GOD@ARV-771(AZO) in PBS at pH 6.5 or 7.4 containing Na₂S₂O₄ as a NTR mimetic. (H) Colloidal stability assessment of HSA-Fc-GOD@ARV-771(AZO) nanoparticles in water, PBS, and cell culture medium over seven days. (**I**) Hemolysis assay evaluating red blood cell membrane integrity following exposure to various concentrations of HSA-Fc-GOD@ARV-771(AZO) nanoparticles.

**Fig. 4:**
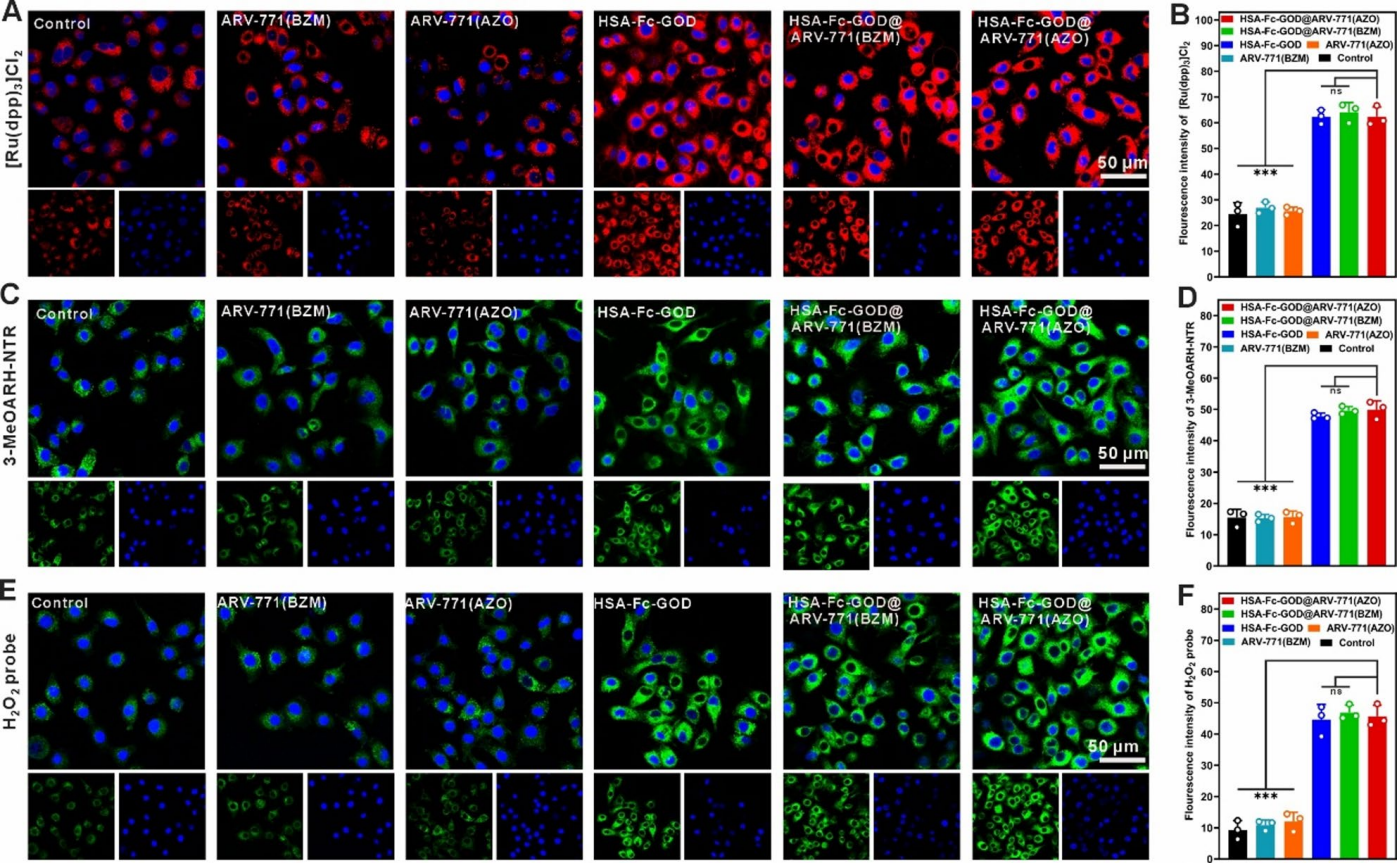
Intracellular hypoxia-responsive cascade by HSA-Fc-GOD@ARV-771(AZO) treatment. CLSM images of A549 lung cancer cells following 12 h treatment with indicated formulations under hypoxic conditions (*n* = 3 independent biological replicates; data are presented as mean ± SD). (**A**) Intracellular oxygen depletion visualized using the oxygen-sensitive phosphorescent probe [Ru(dpp)_3_]Cl_2_ (red fluorescence). (**B**) Mean flourescence intensity of [Ru(dpp)_3_]Cl_2_ shown in (**A**). (**C**) NTR upregulation detected with the enzyme-specific fluorogenic probe 3-MeOARH-NTR (green fluorescence). (**D**) Mean flourescence intensity of 3-MeOARH-NTR shown in (**C**). (**E**) Intracellular H_2_O_2_ accumulation quantified using a green, fluorescent H_2_O_2_-selective probe. (**F**) Mean flourescence intensity of H_2_O_2_-selective probe shown in (**E**). Cell nuclei were counterstained with Hoechst 33,258 (blue). Scale bar, 50 μm. Statistical significance determined by one-way ANOVA with Tukey post hoc test; ***p < 0.001

**Fig. 5:**
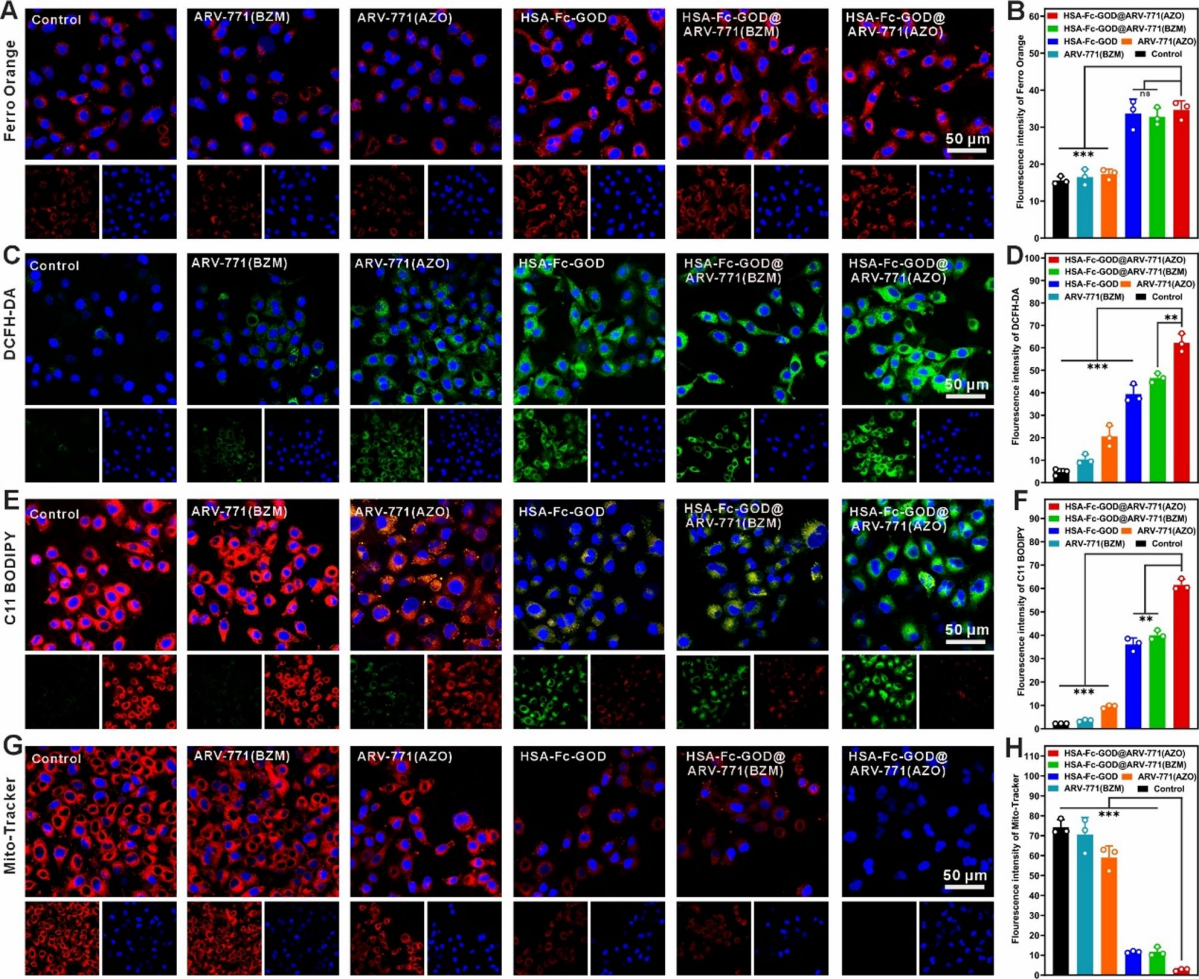
Intracellular ferroptosis cascade visualization in A549 lung cancer cells. CLSM analysis of ferroptotic biomarkers following 12 h treatment with designated formulations under hypoxic conditions (*n* = 3 independent biological replicates; data are presented as mean ± SD). (**A**) Labile ferrous iron accumulation detected with Ferro Orange fluorescent probe (red fluorescence). (**B**) Mean flourescence intensity of Ferro Orange shown in (**A**). (**C**) ROS generation measured using the oxidation-sensitive indicator (green fluorescence). (**D**) Mean flourescence intensity of DCFH-DA shown in (**C**). (**E**) LPO assessed by ratiometric fluorescence shift of C11-BODIPY 581/591 from red (intact lipids) to green (oxidized lipids). (**F**) Mean flourescence intensity of C11-BODIPY 581/591 shown in (**E**). (**G**) Mitochondrial membrane potential collapse visualized with Mito-Tracker Red CMXRos (red fluorescence). (**H**) Mean flourescence intensity of Mito-Tracker shown in (**G**). Cell nuclei were counterstained with Hoechst 33,258 (blue fluorescence). Scale bar, 50 μm. Statistical significance determined by one-way ANOVA with Tukey post hoc test; **p < 0.01, ***p < 0.001

**Fig. 6:**
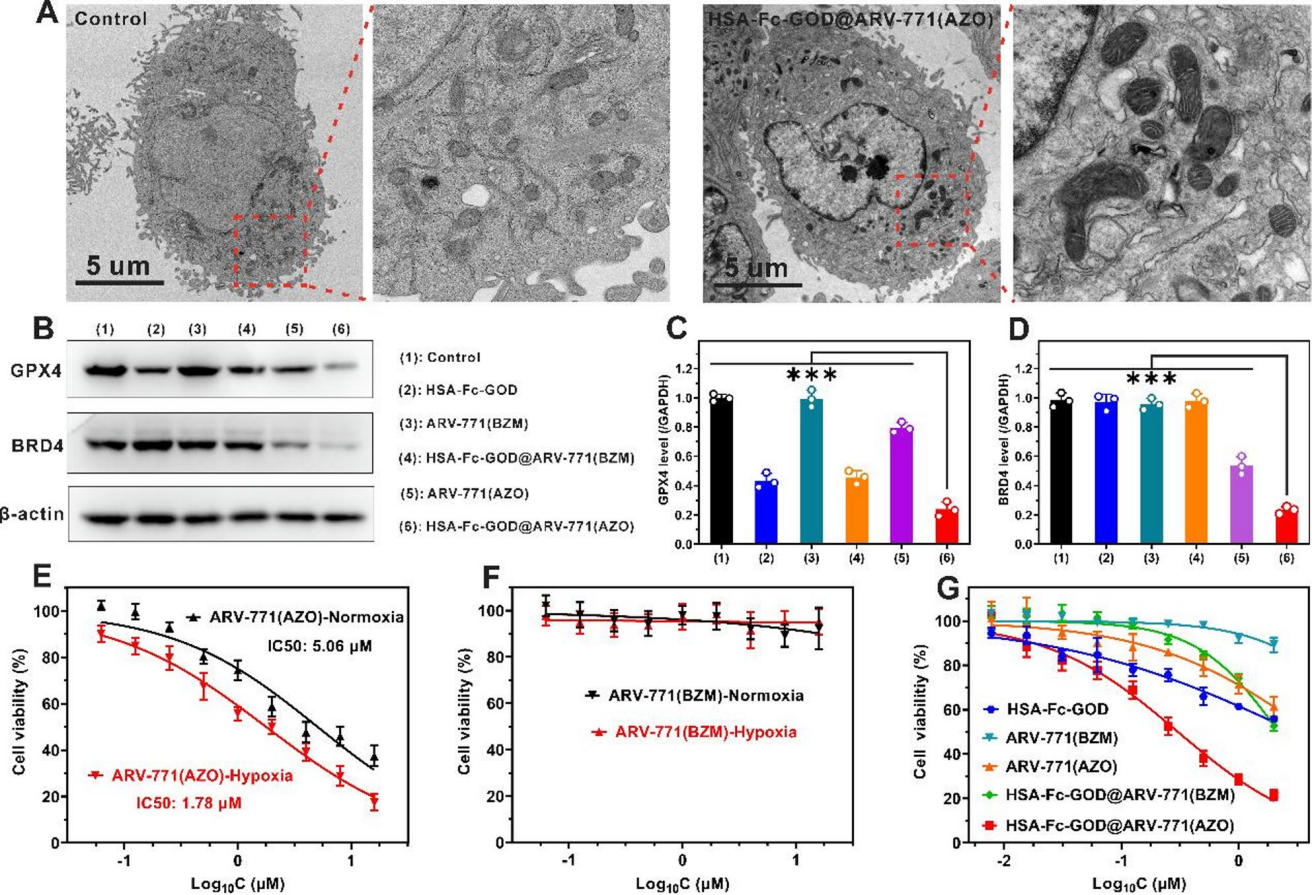
Synergistic in vitro antitumor efficacy through integrated starvation therapy, ferroptosis induction, and PROTAC-mediated protein degradation. (**A**) TEM images of A549 cells following 12 h treatment with culture medium (control) or HSA-Fc-GOD@ARV-771(AZO) under hypoxic conditions (1% O₂). Scale bar, 5 μm. (B) Western blot analysis of GPX4 and BRD4 protein expression in A549 cells treated with indicated formulations for 24 h under hypoxic conditions (1% O₂). (**C, D**) Densitometric quantification of GPX4 (**C**) and BRD4 (**D**) band intensities normalized to β-actin loading control (*n* = 3 independent biological replicates; data are presented as mean ± SD). Statistical significance determined by one-way ANOVA with Tukey post hoc test; ***p < 0.001. (**E, F**) Hypoxia-dependent cytotoxicity of ARV-771(AZO) (**E**) and the non-cleavable control ARV-771(BZM) (**F**) against A549 cells following 48 h incubation under normoxic (21% O₂) versus hypoxic (1% O₂) conditions (*n* = 6 independent experiments; data are presented as mean ± SD). (**G**) Concentration-dependent cell viability of A549 cells treated with various formulations for 48 h under hypoxic conditions (*n* = 6 independent experiments; data are presented as mean ± SD)

**Fig. 7:**
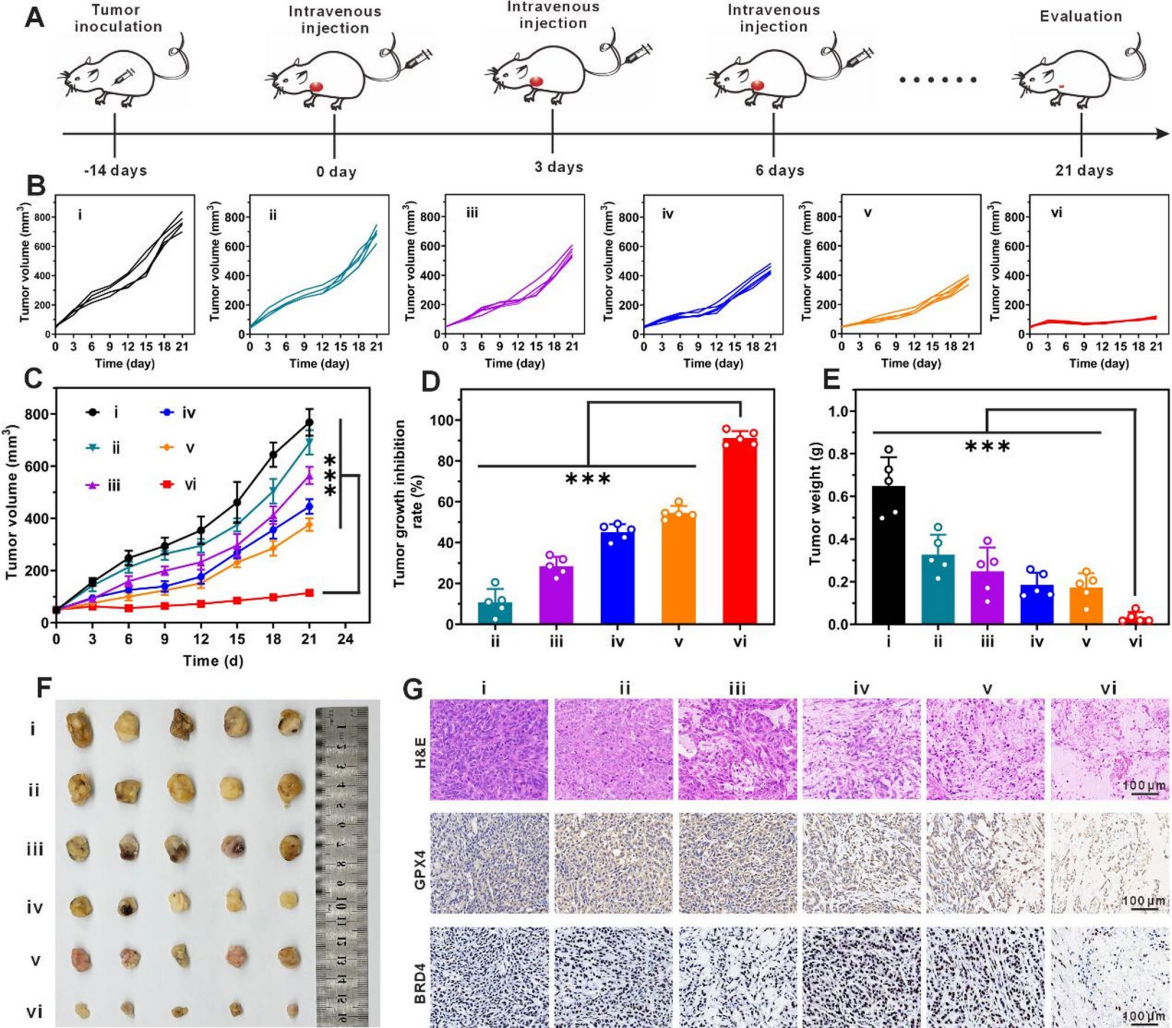
In vivo antitumor efficacy and mechanistic validation in an A549 xenograft model. Female BALB/c nude mice bearing subcutaneous A549 lung cancer xenografts were randomly assigned to six treatment groups (*n* = 5 mice per group) receiving intravenous injections every three days for a total of seven doses. (i) phosphate-buffered saline control, (ii) ARV-771(BZM), (iii) ARV-771(AZO), (iv) HSA-Fc-GOD, (v) HSA-Fc-GOD@ARV-771(BZM), and (vi) HSA-Fc-GOD@ARV-771(AZO). (**A**) Schematic representation of the treatment protocol and experimental timeline. (**B**) Individual tumor growth trajectories for each treatment group over the 21-day study period. (**C**) Mean tumor volume progression for all groups (*n* = 5 mice per group); data are presented as mean ± SD). (**D**) Tumor growth inhibition rates calculated at the experimental endpoint (day 21) relative to phosphate-buffered saline controls (*n* = 5 mice per group); data are presented as mean ± SD. (**E**) Mean tumor weights of excised tissues from each treatment group at study termination (*n* = 5 mice per group); data are presented as mean ± SD). (**F**) Representative photographs of excised tumors from each treatment group at day 21. (**G**) Histopathological and immunohistochemical analysis of tumor sections displaying H&E staining (top row), GPX4 expression (middle row), and BRD4 expression (bottom row). Scale bar, 100 μm. Statistical significance was determined by two-way repeated measures ANOVA with Tukey’s post hoc test for tumor growth curves (**C**), and one-way ANOVA with Tukey’s post hoc test for endpoint analyses (**D** and **E**); ***p < 0.001

The HSA-Fc-GOD@ARV-771(AZO) nanoparticles were subsequently obtained through a hydrophobic drug-induced self-assembly process [36], followed by purification via high-speed centrifugation and ultrafiltration. UV-vis-NIR spectroscopy revealed a distinct ARV-771(AZO) absorption band at 254 nm with a pronounced enhancement in intensity, thereby validating efficient prodrug incorporation (Fig. 2H). The drug loading content of ARV-771(AZO) in the HSA-Fc-GOD@ARV-771(AZO) nanoparticles was determined to be 8.82% (wt%) based on UV-vis spectroscopy analysis. The integration of hydrophobic ARV-771(AZO) drove the hierarchical self-assembly of the HSA-Fc-GOD complexes into uniform nanostructures. TEM imaging revealed monodisperse spherical nanoparticles with an average diameter of approximately 80 nm (Fig. 2I), substantially larger than the ~ 10 nm precursor proteins, thereby demonstrating successful assembly and structural integrity of the nanoplatform.

### Coupled enzymatic activity and Fenton chemistry within the nanoplatform

The therapeutic mechanism of HSA-Fc-GOD@ARV-771(AZO) relies on the catalytic activity of GOD to deplete intratumoral nutrients and oxygen while generating H_2_O_2_ as a substrate for downstream Fenton reactions. We first verified whether the enzymatic activity of GOD was retained after nanoparticle assembly. In the presence of glucose, the dissolved oxygen concentration in the nanoparticle suspension rapidly declined from 8.15 mg/L to nearly zero within 40 min (Fig. 3A), accompanied by a pH decrease from 7.4 to 4.29 due to gluconic acid formation (Fig. 3B). In contrast, no detectable changes were observed in glucose-free buffer, thereby confirming that the catalytic function of GOD remained intact. Quantitative analysis further revealed that H₂O₂ generation increased linearly with glucose concentration (Fig. 3C), demonstrating that glucose oxidation and oxygen consumption were tightly coupled within the nanoplatform.

We next examined whether the Fc moiety could harness this enzymatically produced H₂O₂ to drive iron-mediated Fenton chemistry. Using methylene blue (MB) as a •OH probe [37], nearly complete degradation of MB occurred only in the presence of HSA-Fc-GOD or the complete nanoplatform, whereas isolated components showed negligible activity (Fig. 3D). This observation was further corroborated by electron spin resonance (ESR) spectroscopy, which exhibited a strong signal corresponding to the DMPO/•OH adduct (Fig. 3E) [38], and by a fluorescence assay employing disodium terephthalate (Fig. 3F) [39], which revealed a pronounced increase in •OH production. Collectively, these results confirm that GOD within the nanoparticles effectively catalyzes glucose-dependent H₂O₂ formation, which is subsequently converted into cytotoxic •OH through Fe-mediated Fenton reactions, thereby coupling metabolic starvation with oxidative damage in a single nanoplatform.

### Tumor microenvironment-responsive payload release and biocompatibility

Drug release profiling revealed tumor-relevant responsiveness of the HSA-Fc-GOD@ARV-771(AZO) nanoparticles. At pH 6.5, 51.4% of ARV-771 was released within 24 h, compared with 29.8% at pH 7.4 (Fig. 3G), consistent with protonation of histidine and carboxylate residues on HSA that perturbs hydrophobic pockets and the electrostatic microenvironment, thereby weakening hydrophobic, electrostatic, and hydrogen-bonding interactions, lowering binding affinity, and accelerating desorption from binding sites and the hydrophobic core [40]. As the payload is released, depletion of the hydrophobic core drives supramolecular rearrangement and compaction, yielding nanoparticles with reduced diameters (Fig. S8). In parallel, reductive hypoxia-mimicking conditions employed in the assay (Na₂S₂O₄ as an NTR surrogate) further promote AZO linker cleavage and on-demand prodrug liberation. This pH sensitivity, coupled with the hypoxia-activated linker cleavage, facilitates on-demand prodrug liberation within tumor microenvironments.

HSA-Fc-GOD@ARV-771(AZO) nanoparticles also maintained excellent colloidal stability in water, PBS, and cell-culture medium for one week without aggregation (Fig. 3H), thereby underscoring their physicochemical robustness. Moreover, hemolysis assays revealed less than 5% red-cell disruption even at 1000 µg/mL (Fig. 3I), confirming high hemocompatibility and supporting the feasibility of intravenous administration and systemic tolerability.

### Intracellular hypoxia-responsive therapeutic cascade

To elucidate the intracellular mechanisms underlying the synergistic antitumor activity, we examined the sequential biochemical events in A549 lung cancer cells under hypoxia (1% O₂). Hypoxia mapping using the oxygen-responsive probe [Ru(dpp)₃]Cl₂ revealed markedly enhanced red fluorescence in cells treated with GOD-containing nanoparticles (Fig. 4A, B) [41], indicating pronounced intracellular oxygen depletion. This GOD-induced hypoxia upregulated NTR expression, as visualized by the NTR-specific fluorescent probe 3-MeOARH-NTR (Fig. 4C, D) [42], thereby confirming a hypoxia-driven cascade process in which the nanoplatform exacerbates hypoxia to promote NTR-mediated cleavage of the ARV-771(AZO) prodrug and release of the active PROTAC degrader. These observations establish the foundation of a hypoxia-gated therapeutic cascade wherein GOD-mediated metabolic intervention directly facilitates hypoxia-gated drug activation, thus addressing the intrinsic limitation of suboptimal PROTAC bioavailability through on-demand prodrug conversion within the tumor microenvironment. Given our earlier observation that extracellular GOD catalyzed the consumption of O₂ concomitant with H₂O₂ generation, we next quantified changes in intracellular H_2_O_2_ levels using a green, fluorescent H_2_O_2_-responsive probe. As expected, cells exposed to GOD-containing nanoparticles displayed markedly higher fluorescence than those treated with GOD-free controls (Fig. 4E, F), thereby confirming that the enzymatic activity of GOD significantly elevates intracellular H₂O₂ levels. The elevated H₂O₂ serves as an endogenous oxidant source for Fenton-like reactions, addressing the ROS deficiency characteristic of standard ferroptosis approaches and laying the groundwork for cascade-enhanced therapeutic outcomes.

We next interrogated ferroptosis-related events. Labile iron quantification with FerroOrange revealed substantial Fe²⁺ accumulation in cells treated with iron-containing nanoparticles (Fig. 5A, B) [43]. Together with GOD-mediated H₂O₂ production, this iron pool drove a marked surge in reactive oxygen species (ROS) as measured by DCFH-DA [44], with the highest signal observed in the HSA-Fc-GOD@ARV-771(AZO) group (Fig. 5C, D). The elevated ROS levels can be attributed to BRD4 degradation caused by hypoxia-triggered release of ARV-771, which suppresses GPX4 expression and thereby compromises the glutathione-dependent reduction of lipid hydroperoxides to non-toxic alcohols [45]. This GPX4 deficiency establishes a sustained oxidative cycle in which Fenton-generated •OHs drive sustained LPO in the absence of adequate antioxidant capacity. These data indicate a synergism in which ARV-771-driven BRD4 degradation heightens susceptibility to oxidative stress. Sustained ROS exposure induced extensive LPO, evidenced by the characteristic red-to-green shift of C11-BODIPY fluorescence (Fig. 5E, F) [46], and was accompanied by severe mitochondrial injury as shown by Mito-Tracker staining [47], including loss of membrane potential (Fig. 5G, H) and ultrastructural damage observed by TEM such as mitochondrial shrinkage, cristae disruption, and outer-membrane rupture (Fig. 6A). The observed mitochondrial dysfunction exacerbates the metabolic stress induced by glucose deprivation, thereby establishing a convergent pathway through which starvation therapy and oxidative damage cooperate to precipitate cell death.

To further validate the occurrence of ferroptosis, we examined the expression of GPX4, a key protein intimately associated with ferroptotic cell death. As one of the most critical negative regulators of ferroptosis, GPX4 suppresses this process by catalyzing the glutathione-dependent reduction of lipid hydroperoxides. Consequently, decreased GPX4 expression serves as an important molecular indicator of ferroptosis execution. We found that the HSA-Fc-GOD@ARV-771(AZO) treatment group exhibited the lowest GPX4 expression levels (Fig. 6B, C), which was consistent with the LPO results described earlier. In parallel, we also assessed intracellular BRD4 expression levels through Western blot analysis. ARV-771(BZM) bearing a non-cleavable benzylmethyl linker and its nanoparticle formulation did not alter the abundance of BRD4 because the active PROTAC degrader was not liberated from its prodrug configuration. In contrast, ARV-771(AZO) contains a hypoxia-cleavable AZO group and its nanoparticle formulation reduced intracellular B.

BRD4 abundance through targeted protein degradation (Fig. 6B, D). Notably, the HSA-Fc-GOD@ARV-771(AZO) treatment group displayed substantially lower BRD4 protein expression compared to other groups. This outcome can be attributed to the GOD-mediated O₂ consumption, which intensified the intracellular hypoxic conditions, thereby promoting NTR overexpression and subsequent release of greater amounts of ARV-771, leading to efficient degradation of BRD4 protein within the cells. These findings collectively reveal that hypoxia-triggered reduction of AZO group and ARV-771 release combine with Fenton-catalyzed ferroptosis to generate a synergistic therapeutic axis wherein each mechanism potentiates the others.

### Hypoxia-gated activation and synergistic in vitro cytotoxicity

Encouraged by the preceding cellular mechanistic studies, we evaluated the hypoxia-dependent cytotoxicity of HSA-Fc-GOD@ARV-771(AZO) using MTT assays. Initial assessment of individual prodrugs revealed distinct hypoxia responsiveness profiles. ARV-771(BZM) exhibited negligible cytotoxicity under both normoxic and hypoxic conditions, maintaining 92.3% cell viability at concentrations up to 16 µM (Fig. 6E), whereas ARV-771(AZO) demonstrated marked cytotoxic activity (Fig. 6F). Notably, ARV-771(AZO) displayed 2.85-fold enhanced potency against A549 cells under hypoxic versus normoxic conditions (IC₅₀ = 1.78 µM versus 5.06 µM), attributable to hypoxia-triggered PROTAC liberation. Integration of ARV-771(AZO) into the complete HSA-Fc-GOD nanoplatform yielded the most pronounced antiproliferative activity among all formulations tested, reducing cell viability to 10.8% (Fig. 6G). The enhanced efficacy results from the combination of glucose deprivation, ferroptosis induction, and PROTAC-based degradation. The cytotoxicity data confirm this integrated approach wherein GOD creates metabolic stress and deepens hypoxia to release the prodrug, thereby initiating sequential oxidative injury, and mitochondrial collapse that drive selective tumor cell death.

### Potent in vivo antitumor efficacy via multimodal synergism

We performed anticancer studies in vivo using an A549 xenograft model (Fig. 7A).

Prior to efficacy trials, we evaluated the pharmacokinetic profile. Free ARV-771 was cleared rapidly from the circulation with a half-life of 1.85 h (Fig. S9A and Table S1). In contrast, the HSA-Fc-GOD@ARV-771(AZO) formulation significantly extended the circulation half-life to 7.92 h. The systemic clearance rate was reduced from 2.40 L/h/kg to 0.56 L/h/kg. Consequently, the total systemic exposure (AUC) was enhanced by approximately 4.3-fold. Biodistribution analysis further showed that while free ARV-771 exhibited negligible retention in tumor tissues (Fig. S9B), the nanoparticles showed preferential and sustained accumulation in the tumor site over 24 h (Fig. S9C). This accumulation is driven by the enhanced permeability and retention effect combined with the natural affinity of albumin for gp60 receptors on endothelial cells and SPARC in the tumor extracellular matrix [20].

Throughout the 21-day treatment period, tumor dimensions were measured every three days using digital calipers to calculate tumor volume via the modified ellipsoid formula V = (length × width²)/2 and construct growth curves by plotting volume against time (Fig. 7B, C). Mice treated with HSA-Fc-GOD@ARV-771(AZO) exhibited substantially suppressed tumor growth because of the coordinated integration of GOD-mediated glucose deprivation, ferroptotic oxidative injury, and hypoxia-activated BRD4 degradation. The treatment achieved a tumor growth inhibition rate of 94.3% (Fig. 7D), significantly outperforming the efficacy observed in mice treated with HSA-Fc-GOD (61.7%), HSA-Fc-GOD@ARV-771(BZM) (54.7%), or the free prodrugs. The marked superiority of HSA-Fc-GOD@ARV-771(AZO) stems from albumin-mediated tumor targeting and pH-triggered payload release, whereupon GOD-induced metabolic stress and hypoxia activate NTR-mediated PROTAC liberation for BRD4 degradation that suppresses GPX4 and sensitizes cells to iron-catalyzed ferroptotic death. At the study endpoint (day 21), mice were sacrificed, and the tumors were excised, weighed, and photographed (Fig. 7E, F). Consistent with the growth curve data, the mean weight of tumors from the HSA-Fc-GOD@ARV-771(AZO) group was substantially lower than that of other groups.

To elucidate the in vivo mechanism of action, the excised tumor tissues were subjected to histological and immunohistochemical analysis. Histological examination showed extensive necrosis and reduced tumor cellularity (Fig. 7G), consistent with the catastrophic cellular damage expected from the convergent action of starvation, oxidative stress, and proteolytic degradation. Immunohistochemistry confirmed the dual downregulation of BRD4 and GPX4, thereby validating the simultaneous engagement of PROTAC and ferroptotic cell-death pathways. The pronounced reduction in BRD4 expression within the tumor tissue confirmed efficient hypoxia-activated PROTAC liberation and target engagement in vivo, while the parallel suppression of GPX4 validated the execution of ferroptotic cell death as a dominant mechanism underlying the observed therapeutic response. The spatial colocalization of BRD4 depletion and GPX4 downregulation implies a mechanistic dependency. Since BRD4 functions as a transcriptional co-activator for antioxidant defense genes, its degradation likely induces transcriptional repression of GPX4, thereby downregulating GPX4 protein expression [48]. This reduction in GPX4 protein levels impairs the cell’s ability to reduce toxic lipid hydroperoxides to benign alcohols, thereby sensitizing the tumor cells to ferroptotic death.

We comprehensively evaluated the systemic biosafety profile to rule out off-target toxicity particularly GOD-induced hypoglycemia. Treated mice maintained stable body weights and blood glucose levels between 6 and 7 mmol/L throughout the study which confirms that vital organs such as the brain were spared from metabolic stress (Fig. S10, 11). This safety profile was corroborated by serum biochemistry analysis which showed no abnormalities in cardiac hepatic or renal function markers alongside histological assessments revealing no pathological changes in major organs (Fig. S12, 13). Notably the physiological oxygenation and pH of hepatic tissue prevent the activation of the prodrug despite the natural accumulation of albumin carriers in the liver. The cleavage of the azobenzene linker is strictly confined to the deep hypoxia of the tumor microenvironment which maximizes the therapeutic index while preserving systemic health.

Despite the therapeutic potential of ferroptosis there are significant hurdles for its clinical translation. A primary concern is the risk of off-target iron accumulation which can trigger severe oxidative damage in healthy organs. Other barriers include the scarcity of validated clinical biomarkers and the challenges associated with the large-scale manufacturing of complex nanomedicines. Our study attempts to address these limitations by employing a precise activation strategy. The albumin-mediated delivery concentrates the drug in the tumor while the hypoxia-responsive design ensures that iron-catalyzed reactive oxygen species generation occurs strictly within the tumor microenvironment. This spatial control minimizes systemic exposure and prevents iron dysregulation in normal tissues. The negligible toxicity observed in our hemolysis assays and histological analysis of major organs supports the potential of this strategy to overcome current safety barriers.

Beyond the direct cytotoxic effects, we postulate that ferroptosis functions as a distinct immunogenic event that transcends silent apoptotic cell death. The catastrophic rupture of the plasma membrane releases a spectrum of damage-associated molecular patterns including HMGB1 and ATP while promoting the surface exposure of calreticulin [49]. These immunomodulatory signals are critical for the recruitment and maturation of dendritic cells which subsequently prime cytotoxic CD8 + T lymphocytes to infiltrate the tumor parenchyma. This immunological awakening suggests that our nanoplatform could potentially reverse the immunosuppressive nature of the tumor microenvironment by converting cold tumors into hot phenotypes. Furthermore we preemptively addressed the challenge of adaptive resistance where malignant cells upregulate antioxidant networks such as the NRF2-SLC7A11 axis to mitigate oxidative stress [50]. The PROTAC component counteracts this by degrading BRD4 which is enriched at super-enhancers driving these survival programs [51]. Consequently, the depletion of BRD4 collapses the transcriptional machinery responsible for glutathione synthesis and metabolic rewiring which ensures that the tumor cells remain vulnerable to the therapeutic LPO.

## Conclusion

This study presents a microenvironment-responsive albumin nanoplatform that orchestrates synergistic antitumor therapy. It integrates metabolic starvation with ferroptosis induction and targeted protein degradation. The HSA-Fc-GOD@ARV-771(AZO) system addresses fundamental limitations hindering PROTAC and ferroptosis clinical translation by establishing a hypoxia-responsive therapeutic cascade. The acidic tumor pH triggers nanoparticle disassembly and initial payload release. The released GOD then depletes oxygen and glucose while generating H₂O₂. This process induces nutritional deprivation and exacerbates hypoxia. The heightened hypoxia upregulates nitroreductase to cleave the AZO linker and liberate the PROTAC. The enzymatically produced H₂O₂ undergoes ferrocene-catalyzed Fenton reactions. This generates •OHs that trigger LPO. Concurrently, PROTAC-mediated BRD4 degradation suppresses GPX4 expression. This sensitizes cells to ferroptotic death. This convergent mechanism achieved a 94.3% tumor growth inhibition rate. We observed no significant systemic toxicity. Future studies could explore combining this cascade with immunotherapy to leverage ferroptosis-induced immunogenicity. Alternatively, radiotherapy could be used to further amplify the hypoxic trigger. The platform establishes a generalizable framework for precision nanomedicines that leverage tumor microenvironment characteristics for conditional drug activation. This provides a translatable prototype for next-generation cancer therapeutics that unite multimodal mechanisms within unified delivery systems.

## Supplementary Information

Supplementary Material 1

## Data Availability

Data will be made available on request.
