# Supplementary material for: Dual tumor microenvironment-responsive albumin nanoplatform integrates conditional PROTAC activation with starvation and ferroptosis for synergistic cancer therapy
Source: J Nanobiotechnology. 2026 Mar 9;24:367. doi: 10.1186/s12951-026-04266-9 (PMC13085391; doi:10.1186/s12951-026-04266-9)
Supplement: Supplementary file 1 — Supplementary Material 1 [file 12951_2026_4266_MOESM1_ESM.docx]

**Dual tumor microenvironment-responsive albumin nanoplatform integrates conditional PROTAC activation with starvation and ferroptosis for synergistic cancer therapy**

**Authors:**

Lingting Lin^a^, Binyu Chen^a^, Yourui Yang^a^, Ruocheng Deng^a^, Wenfei Niu^a^, Jian Liu^a^*, Wei Xu^a,b^*, Hua Li^a^*

**Affiliations:**

^a^ Institute of Structural Pharmacology & TCM Chemical Biology, Fujian Key Laboratory of Chinese Materia Medica, College of Pharmacy, Fujian University of Traditional Chinese Medicine, Fuzhou 350122, China

^b^ Fujian Health College, Fuzhou 350101, China

**Fig. S1.** Synthesis of ARV-771(AZO) and ARV-771(BZM). (a) i: AcOH; ii: 4-nitrophenyl chloroformate, Pyr, Ar, DCM, room temperature, 2 h; iii: ARV-771, DMAP, TEA, DCM, room temperature, 24 h; (b) iv: 4-nitrophenyl chloroformate, Pyr, Ar, DCM, room temperature, 2 h; v: ARV-771, DMAP, TEA, DCM, room temperature, 24 h.


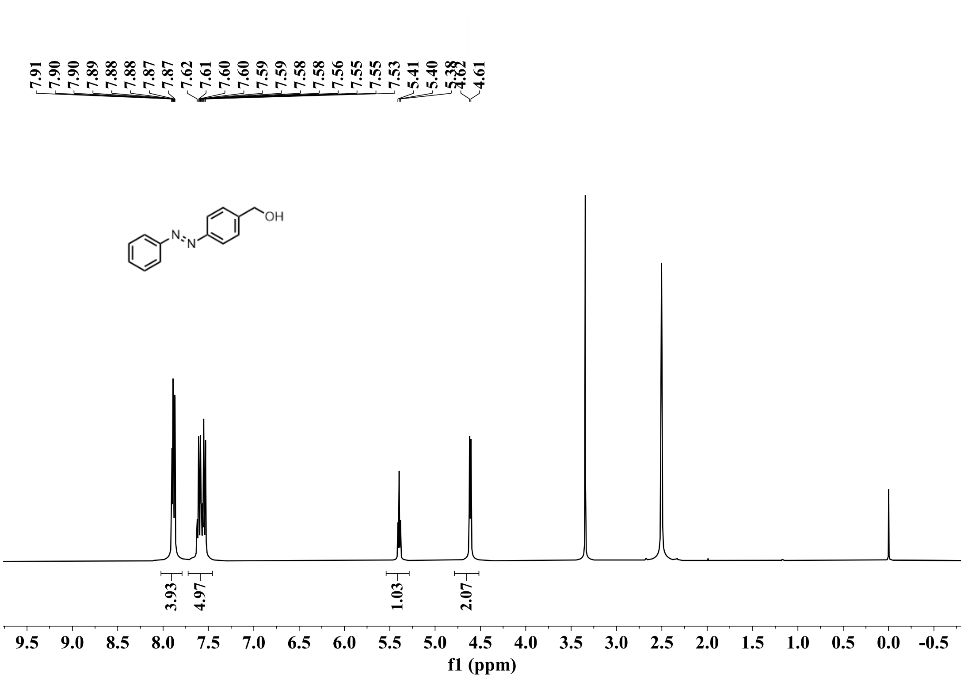


**Fig. S2.** ^1^H NMR spectrum of compound 1.


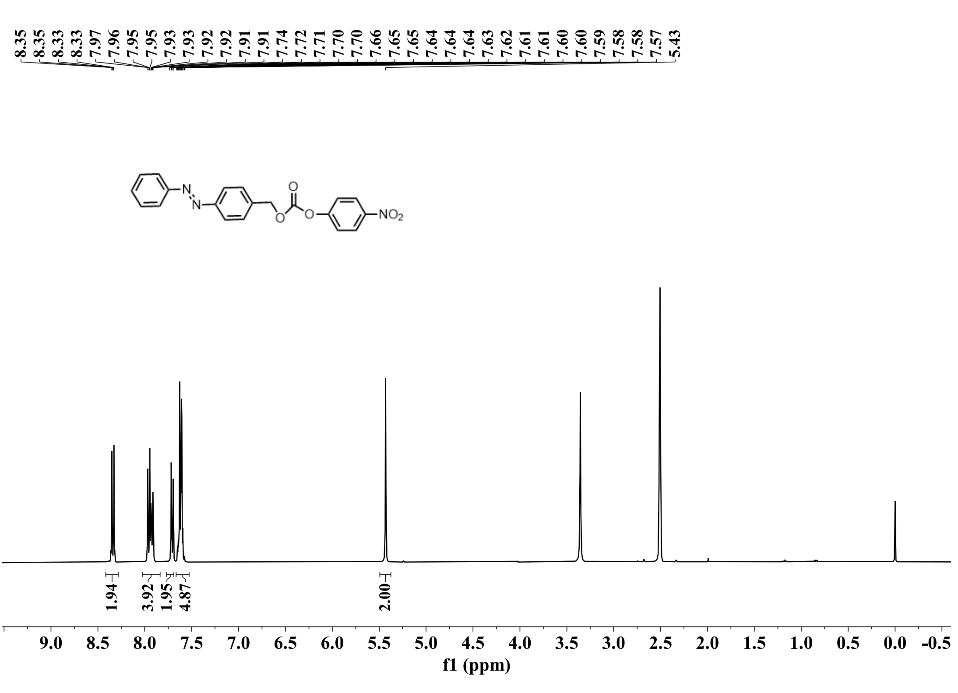


**Fig. S3.** ^1^H NMR spectrum of compound 2.


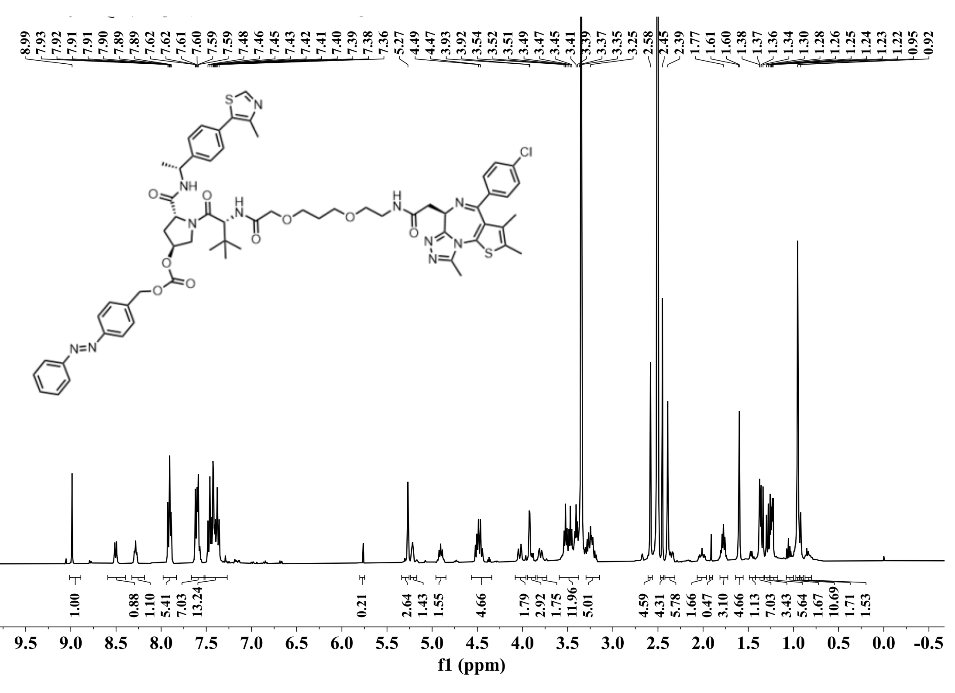


**Fig. S4.** ^1^H NMR spectrum of ARV-771(AZO).

**Fig. S5.** ESI-HRMS of ARV-771(AZO).


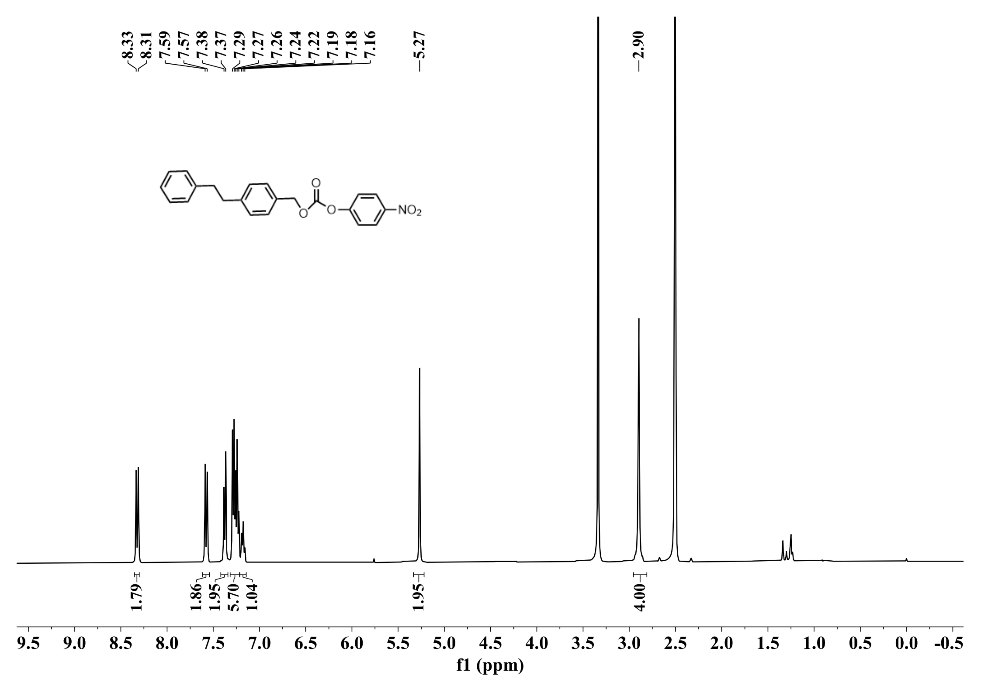


**Fig. S6.** ^1^H NMR spectrum of compound 3.


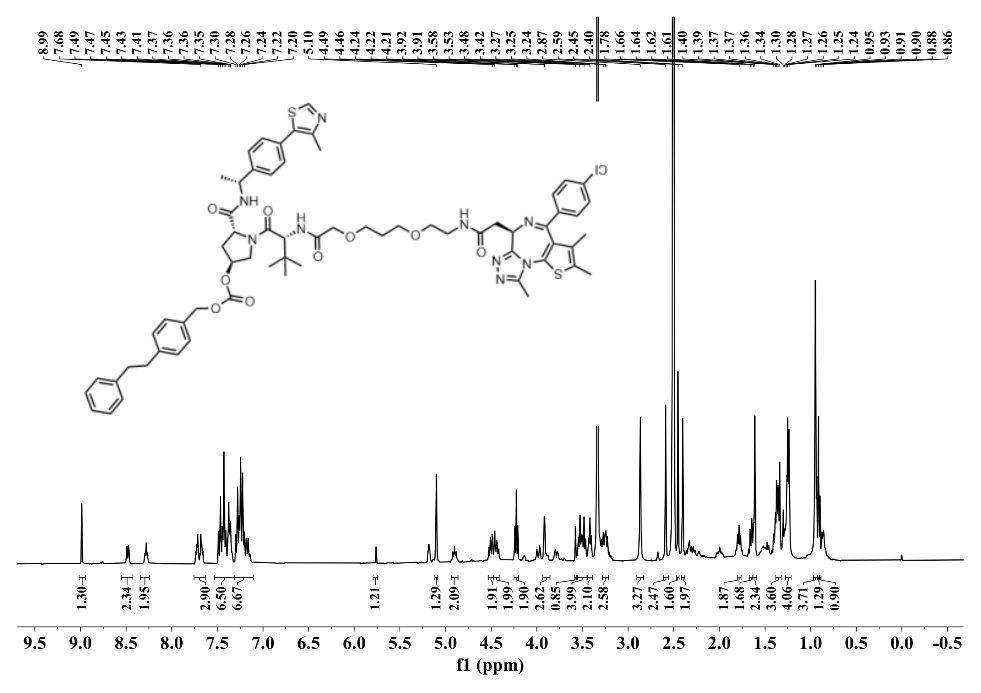


**Fig. S7.** ^1^H NMR spectrum of ARV-771(BZM)


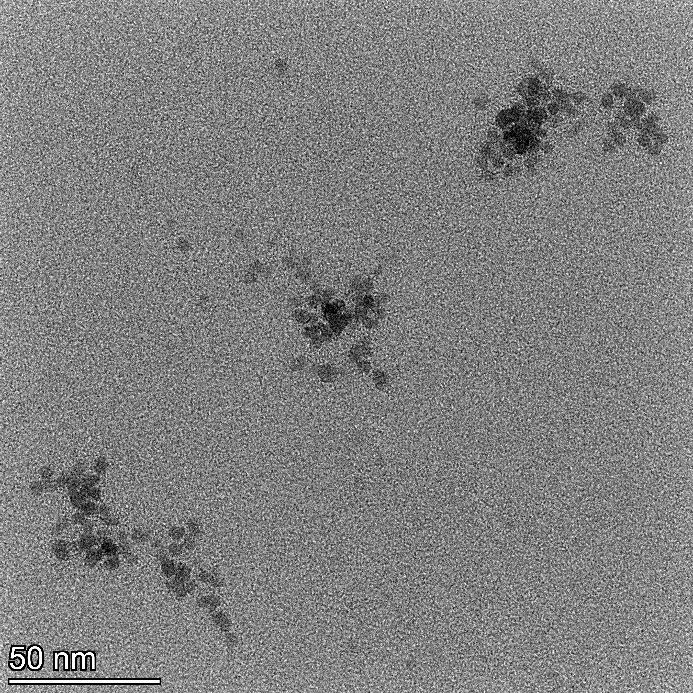


**Fig. S8.** TEM image of HSA-Fc-GOD@ARV-771(AZO) nanoparticles in PBS (pH 6.5). Scale bar, 50 nm.


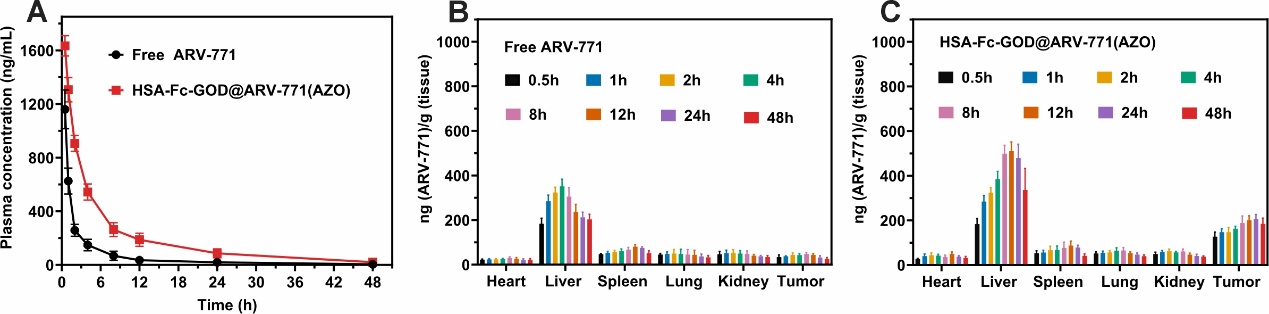


**Fig. S9.** (A) Plasma concentration-time profiles of ARV-771 in tumor-bearing mice following intravenous administration of free ARV-771 and the HSA-Fc-GOD@ARV-771(AZO) conjugate. Biodistribution of ARV-771 in major organs (heart, liver, spleen, lung, kidney) and tumors at indicated time points after treatment with free ARV-771 (B) or HSA-Fc-GOD@ARV-771(AZO) (C). Data are presented as mean ± SD (n = 3). When tumors reached approximately 250 mm³, BALB/c nude mice were randomly divided into two groups (n=3). Mice received an intravenous injection of either free ARV-771 ([ARV-771] = 3.75 mg/kg) or HSA-Fc-GOD@ARV-771(AZO) ([HSA-Fc-GOD] = 10 mg/kg, [ARV-771(AZO)] = 4.65 mg/kg. Three mice from each group were sacrificed at 0.25, 0.5, 1, 2, 4, 6, 12, 24, and 48 h after administration. Approximately 0.8 mL of blood was collected via cardiac puncture into tubes containing heparin sodium. The animals were subsequently perfused with saline to remove residual blood from the tissues. Major organs including the heart, liver, spleen, lung, and kidney were harvested along with the tumors. Plasma was separated by centrifugation and treated with Na₂S₂O₄ (8 mM) to reduce the AZO bond and release the parent ARV-771. A 200 μL aliquot of the treated plasma was mixed with 0.05 μg of the internal standard Tanshinone IIA and extracted with 3 mL of ethyl acetate. The supernatant was dried at 45 °C and redissolved in 100 μL of methanol. The final solution was centrifuged and analyzed using UPLC-MS/MS to determine the concentration of ARV-771.


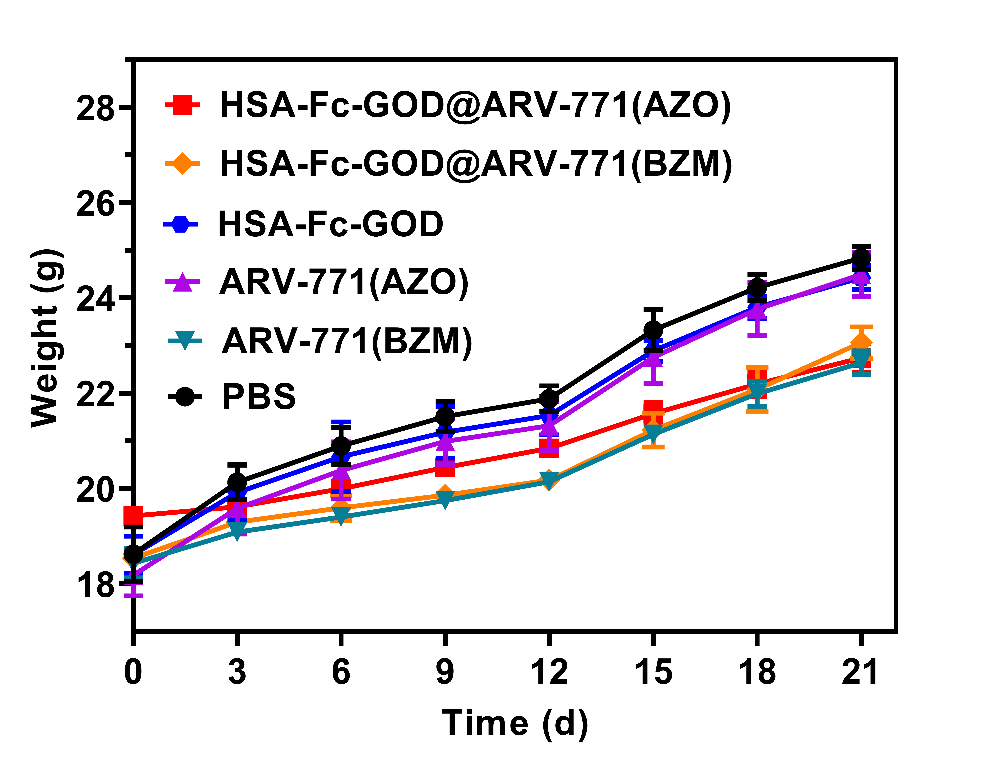


**Fig. S10.** Body weight changes in nude mice in each group over 21 days.


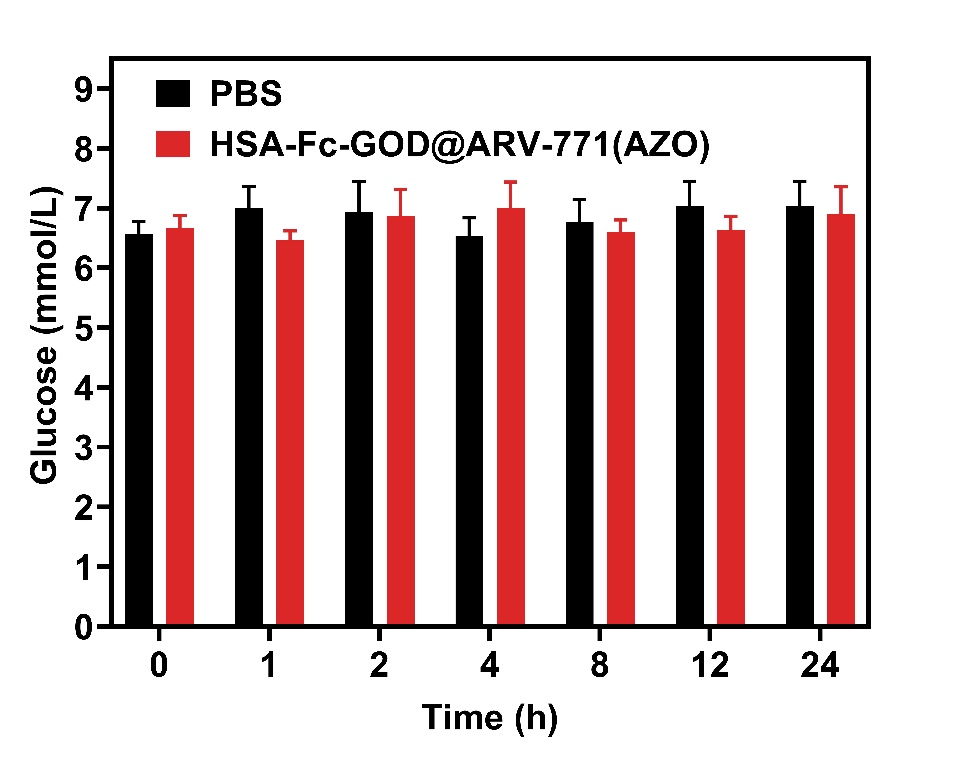


**Fig. S11.** Blood glucose levels of mice recorded at different time points after intravenous injection of PBS or HSA-Fc-GOD@ARV-771(AZO). Data are presented as mean ± SD (n = 3). We investigated the fluctuation of systemic glucose levels to evaluate the biosafety of the starvation therapy. The tumor-bearing mice were randomly assigned to the PBS control group or the treatment group. The mice were intravenously injected with HSA-Fc-GOD@ARV-771(AZO). The administered dosage was equivalent to 10 mg/kg of HSA-Fc-GOD and 4.65 mg/kg of ARV-771(AZO). We monitored the blood glucose concentration at 0, 1, 2, 4, 8, 12, and 24 h post-injection. Blood samples were collected at each time point. The glucose levels were measured using a commercial glucometer.


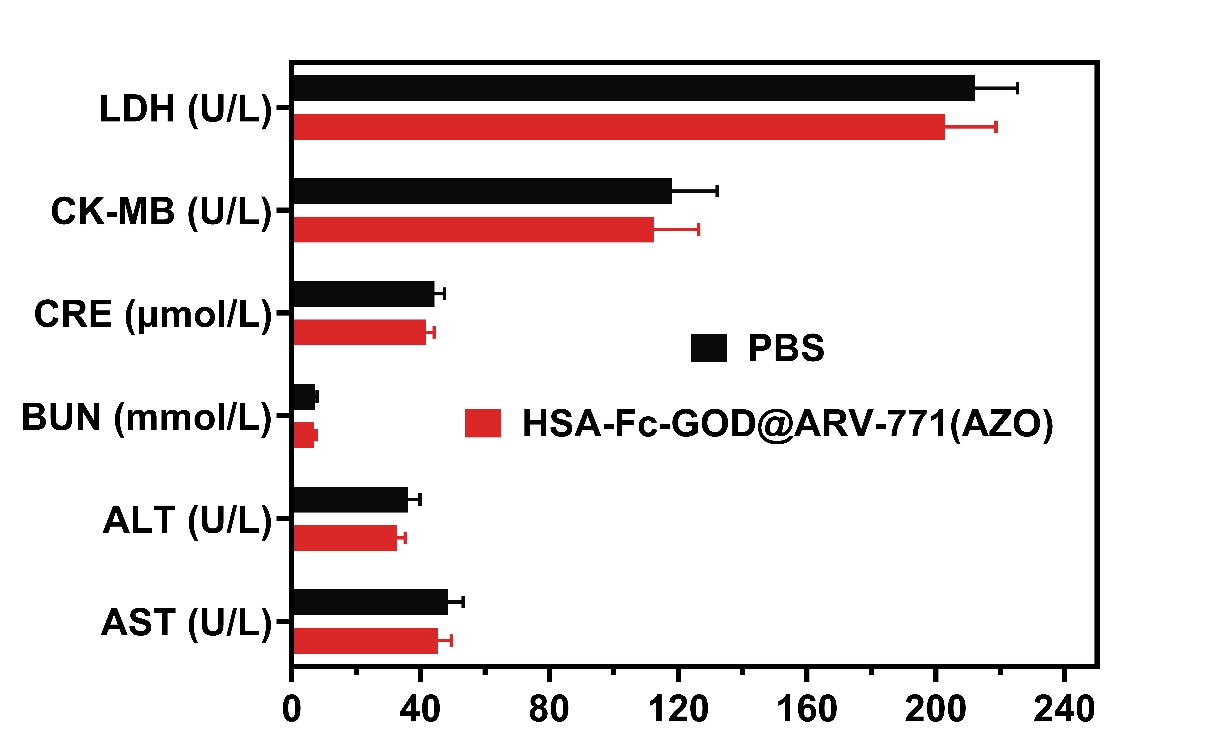


**Fig. S12.** Serum biochemistry analysis of treated mice including liver function markers (ALT and AST), kidney function markers (BUN and CRE), and cardiac function markers (CK-MB and LDH). Data are presented as mean ± SD (n = 3). The mice received the treatment for 21 days. The dosage was normalized to 10 mg/kg of HSA-Fc-GOD and 4.65 mg/kg of ARV-771(AZO). At the end of the therapeutic period, we collected blood samples from the mice and isolated the serum by centrifugation. We evaluated liver function by measuring alanine aminotransferase and aspartate aminotransferase levels. Kidney function was determined by analyzing blood urea nitrogen and creatinine. We also detected creatine kinase-MB and lactate dehydrogenase to examine cardiac health. All biochemical parameters were quantified using standard assay kits.


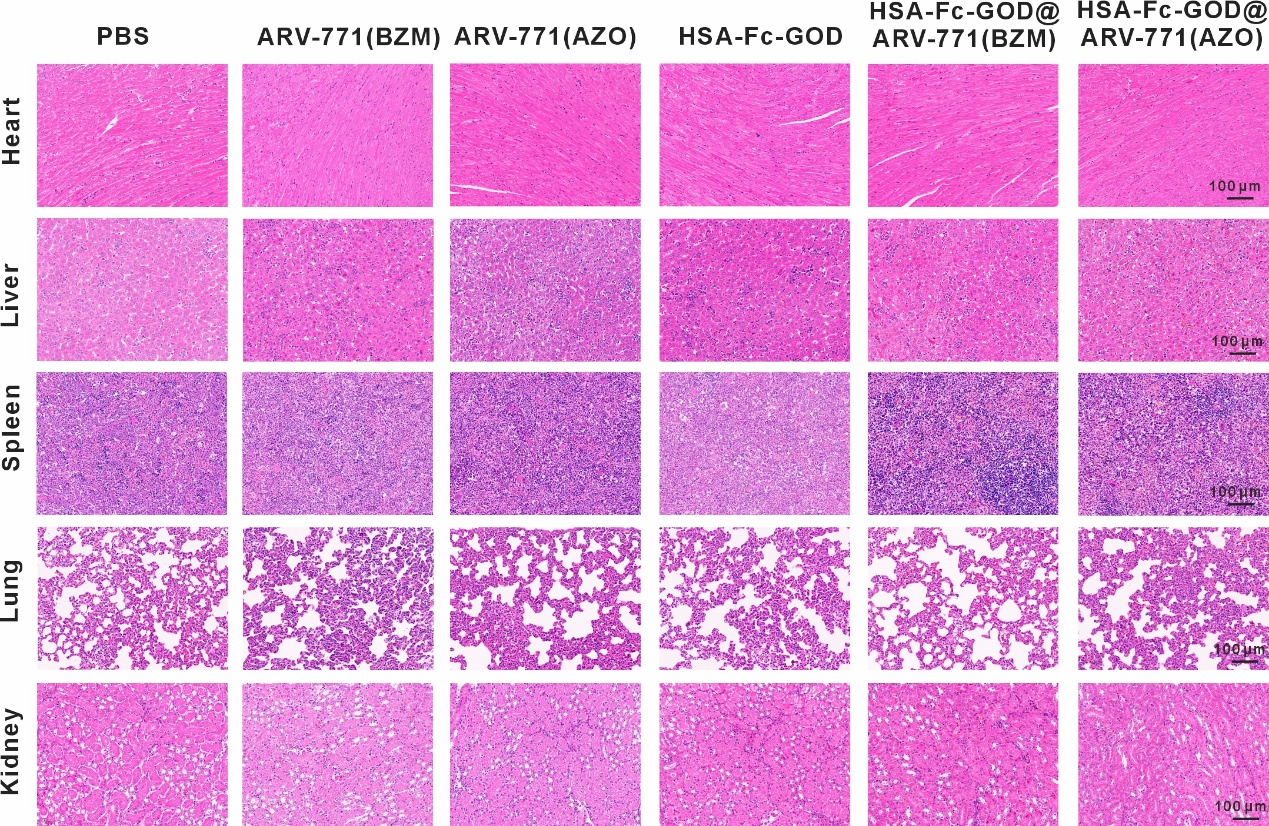


**Fig. S13.** Images of H&E-stained major organs collected from mice of each group after 21 days.

**Table S1.** Pharmacokinetic parameters of Free ARV-771 and HSA-Fc-GOD@ARV-771(AZO) in rats after intravenous injection (n=3, mean ± SD).

| Parameters | Unit | Free ARV-771 | HSA-Fc-GOD@ARV-771 |
| --- | --- | --- | --- |
| AUC_(0–t)_ | ng·h/mL | 2056.4 ± 185.2 | 8645.8 ± 520.5*** |
| AUC _(0–∞)_ | ng·h/mL | 2085.1 ± 190.5 | 8910.2 ± 610.3*** |
| t_1/2_ | h | 1.85 ± 0.42 | 7.92 ± 1.15** |
| MRT_(0–t)_ | h | 2.89 ± 1.56 | 9.02 ± 1.18** |
| MRT _(0–∞)_ | h | 3.24 ± 0.55 | 9.85 ± 1.20** |
| CL | L/h/kg | 2.40 ± 0.21 | 0.56 ± 0.04*** |
| C_max_ | ng/mL | 1160.0 ± 144.2 | 1633.3 ± 76.4* |

Abbreviations: AUC, area under the plasma concentration-time curve; t1/2, elimination half-life; MRT, mean residence time; CL, clearance; Cmax, maximum plasma concentration.

Statistical significance was calculated using Student’s t-test (*p < 0.05, **p < 0.01, ***p < 0.001).
